# Supplementary material for: Degradation Kinetics of Inverted Perovskite Solar Cells
Source: Sci Rep. 2018 Apr 13;8:5977. doi: 10.1038/s41598-018-24436-6 (PMC5899167; doi:10.1038/s41598-018-24436-6)
Supplement: Supplementary file 1 — Supporting Information [file 41598_2018_24436_MOESM1_ESM.pdf]

Supporting Information for: Degradation Kinetics of Inverted Perovskite Solar Cells

Mejd Alsari[1*], Andrew J. Pearson[1], Jacob Tse-Wei Wang[2,3], Zhiping Wang[2], Augusto Montisci[4], Neil C. Greenham[1], Henry J. Snaith[2], Samuele Lilliu[5,6], Richard H. Friend[1]

[1] Cavendish Laboratory, University of Cambridge, CB30HE Cambridge, UK
[2] Clarendon Laboratory, Department of Physics, University of Oxford, OX1 3PU Oxford, UK
[3] CSIRO Energy, Mayfield West, NSW 2304, Australia
[4] University of Cagliari, Dept. of Electrical and Electronic Engineering, 09123 Cagliari, Italy
[5] Department of Physics and Astronomy, University of Sheffield, S3 7RH Sheffield, UK
[6] The UAE Centre for Crystallography, UAE

* Correspondence to: ma671@cam.ac.uk (or mejd.alsari@gmail.com)

Materials and Methods

Perovskite Layer Preparation

$CH_3NH_3PbI_{3-x}Cl_x$ (MAPIC). Methylammonium iodide (MAI) (Dyesol) and $PbCl_2$ (98% Aldrich) (3:1 molar ratio) were dissolved in anhydrous N,N-dimethylformamide (DMF) with final concentrations of 38 wt%. This precursor solution was spincoated at 2000 rpm for 45 s on the hole-transport-layers (HTLs), then annealed in a nitrogen-filled glovebox at 100°C for 90 minutes to produce a ~500 nm thick perovskite layer.

$CH_3NH_3PbI_3$ (MAPI). MAI (Dyesol) and PbI_2 (Aldrich) (3:1 molar ratio), were dissolved in anhydrous DMF with final concentrations of 30 wt% and with the addition of hypophosphorous acid (3 µl ml^{-1}). This precursor solution was spincoated at 2000 rpm for 45 s on the HTL. The substrate was dried for 10 min in a nitrogen-filled glovebox, followed by annealing at 100 °C for 5 min to produce a ~500 nm thick perovskite layer.

$FA_{0.83}Cs_{0.17}Pb(I_{0.8}Br_{0.2})_3$ (mixed-cation). FAI, CsI, $PbBr_2$, and PbI_2 (1:0.2:0.84:0.36 molar ratio), were dissolved in anhydrous DMF:DMSO at 4:1 (v:v) at (1.4 M). This precursor solution was spincoated with a two steps program at 1000 and 6000 rpm for 10 and 30 s respectively. During the second step, 100 µL of Anisole was dripped on the spinning substrate 5 s prior to the end of the program. A ~500 nm thick perovskite layer was produced by annealing the substrate in a nitrogen-filled glovebox at 100 °C for 60 min.

Device Fabrication

All devices fabricated here have the following structure: fluorine-doped tin oxide (FTO)/ poly(3,4-ethylenedioxythiophene) polystyrene sulfonate (PEDOT:PSS)/ poly(N,N'-bis-4-butylphenyl-N,N'-bisphenyl) benzidine (polyTPD)/ perovskite/ phenyl-C61-butyric acid methyl ester (PCBM)/ bathocuproine (BCP)/ Au.[1] A bilayer of PEDOT: PSS and poly-TPD are employed as the hole-transport-layers (HTLs). A piranha-cleaned and patterned FTO substrate was spincoated with PEDOT:PSS. About 20 nm of polyTPD was deposited by spin-coating a hot solution (warmed to 110 °C on a hot plate) containing (1 mg mL^{-1}) poly-TPD (1-Material) and (0.2 mg mL^{-1}) F4-TCNQ (Sigma) in toluene at 2000 rpm for 30 s on the substrate followed by annealing in ambient air at 110 °C for 10 minutes. Then the perovskite layers were prepared following the description above. For the electron-transporting layer (ETL), PCBM (dissolved in DCB, 30 mg ml^{-1}) was spin-coated on top of the perovskite films at 1000 rpm for 30 s. Followed by the deposition of a thin (5 nm) buffer layer of bathocuproine (BCP, 98% Alfa Aesar, 0.5 mg ml^{-1} in IPA) by spinning at 6000 rpm for 20 s. Finally, devices were completed by thermally evaporating ~50nm thick Au contacts. The unencapsulated devices were immediately stored in nitrogen until the *in-situ* and *in-operando* measurements.

In-Situ Current-Voltage, Transient Photocurrent, and Transient Photovoltage Measurements

IV sweeps. To minimize device exposure to air, the atmospheric chamber was brought into a nitrogen-filled glovebox for sample mounting. Continuous dry nitrogen (~10ppm H_2O, ~5ppm O_2) flow was supplied to the chamber and filtered (SGT Super Clean) to minimise residual oxygen, moisture and hydrocarbon content. To monitor oxygen and water content in the chamber we used a gas analyser (Rapidox 3100D – Cambridge Sensotec) connected to the chamber's exit line. A Newport Solar Simulator with an output of one sun (AM 1.5 G) was used for illuminating the entire sample area. A Keithley 2636 SMU was used to collect the IV data. The active area is undefined as devices could not be masked due to the chamber geometry. Therefore PCE and J_{sc} data are normalized throughout the text.

TPC and TPV. For transient measurements (conducted *in-situ*, i.e. in sequence with the IV scans), a 465 nm LED (LED465E, Thor Labs) powered by an Agilent 33500B wavefunction generator with a low-noise PSU was used as the light source. A set of lenses were used to focus the incident light from the pulse light source onto a single pixel of the solar cell. The transient data was recorded with a Tektronix DPO 3032 oscilloscope. The oscilloscope input impedance was set to 50 Ω for the transient photocurrent (TPC) measurements and to 1 MΩ for the transient photovoltage (TPV) measurements with a trans-impedance amplifier. Here, two rectangular pulses from an LED lasting 400 or 100 µs were used for TPC and TPV measurements, respectively. to probe the solar cells. During TPV measurements an extra white light bias from the solar simulator was used to illuminate the solar cells. A LabView VI software was used for instrument control and data acquisition. Data analysis was performed with a MATLAB software.

Devices Metrics (As-Prepared Devices)

Table S 1 | Inverted $CH_3NH_3PbI_{3-x}Cl_x$ (MAPIC) solar cell. Pixel 6 was used for the stability measurements discussed here.

Sweep Direction	J_{sc} [mA cm^{-2}]	PCE [%]	Voc [V]	FF	Pixel no.
Reverse	-18.82	8.96	0.89	0.54	1
Forward	-18.71	9.39	0.89	0.57	1
Reverse	-19.31	9.84	0.92	0.56	2
Forward	-19.16	9.35	0.89	0.56	2
Reverse	-20.28	11.49	0.92	0.63	3
Forward	-20.19	10.98	0.92	0.60	3
Reverse	-18.92	6.83	0.83	0.44	4
Forward	-18.91	6.44	0.83	0.42	4
Reverse	-19.93	11.46	0.95	0.62	5
Forward	-19.90	10.85	0.92	0.60	5
Reverse	-20.06	11.96	0.95	0.64	6
Forward	-19.81	11.33	0.92	0.63	6
Reverse	-19.32	5.17	0.77	0.35	7
Forward	-19.25	4.81	0.74	0.34	7
Reverse	-18.93	11.08	0.95	0.63	8
Forward	-19.01	10.43	0.95	0.59	8
Mean	**-19.41**	**9.40**	**0.89**	**0.54**	
Std	0.52	2.27	0.06	0.10	

Table S 2 | Inverted $CH_3NH_3PbI_3$ (MAPI) solar cell. Pixel 1 was used for the stability measurements discussed here.

Sweep Direction	J_{sc} [mA cm^{-2}]	PCE [%]	Voc [V]	FF	Pixel no.
Reverse	-19.34	15.43	1.10	0.73	1
Forward	-19.18	14.67	1.10	0.70	1
Reverse	-17.35	1.24	0.27	0.27	2
Forward	-17.23	1.21	0.27	0.26	2
Reverse	-19.44	14.21	1.10	0.66	3
Forward	-19.33	13.39	1.07	0.65	3
Reverse	-19.35	7.63	1.04	0.38	4
Forward	-19.14	7.12	1.01	0.37	4
Reverse	-19.26	3.54	0.66	0.28	5
Forward	-19.02	3.43	0.63	0.29	5
Reverse	-17.48	3.23	0.63	0.30	6
Forward	-17.25	3.12	0.63	0.29	6
Mean	**-18.62**	**7.35**	**0.79**	**0.43**	
Std	0.92	5.34	0.31	0.18	

Table S 3 | Inverted $FA_{0.83}Cs_{0.17}Pb(I_{0.8}Br_{0.2})_3$ (mixed-cation) solar cells. Pixel 2 was used for the stability measurements discussed here.

Sweep Direction	J_{sc} [mA cm^{-2}]	PCE [%]	Voc [V]	FF	Pixel no.
Reverse	-22.69	18.45	1.10	0.74	2
Forward	-22.58	15.96	1.07	0.66	2
Reverse	-22.45	18.04	1.10	0.73	3
Forward	-22.25	15.35	1.07	0.64	3
Reverse	-21.95	17.98	1.10	0.74	4
Forward	-21.83	15.37	1.07	0.66	4
Reverse	-22.59	17.79	1.10	0.72	5
Forward	-22.38	15.34	1.07	0.64	5
Reverse	-22.75	18.04	1.07	0.74	6
Forward	-22.39	15.52	1.07	0.65	6
Reverse	-20.41	16.53	1.07	0.76	7
Forward	-20.25	13.89	1.07	0.64	7
Mean	**-22.04**	**16.52**	**1.08**	**0.69**	
Std	0.81	1.43	0.01	0.05	

Current Voltage (IV)

Original IV Data

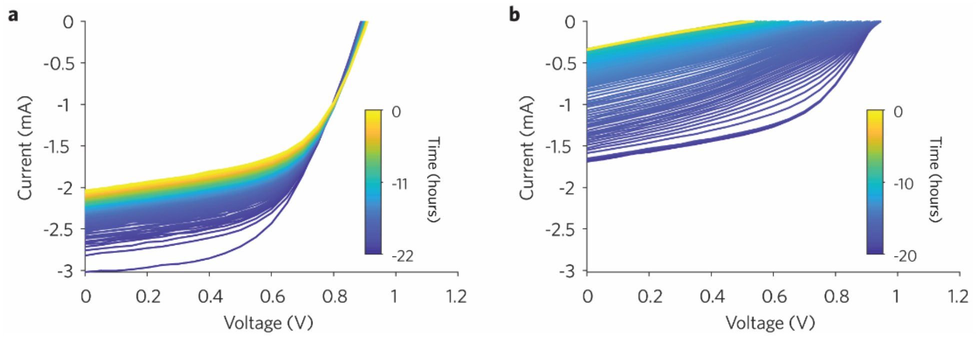

Figure S1 | Current-voltage reverse sweeps of inverted $CH_3NH_3PbI_{3-x}Cl_x$ (MAPIC) solar cell under continuous illumination and dry N_2 [Time (hours) < 0] (a) and dry N_2 (99%) with O_2 (1%) [Time (hours) > 0] (b).

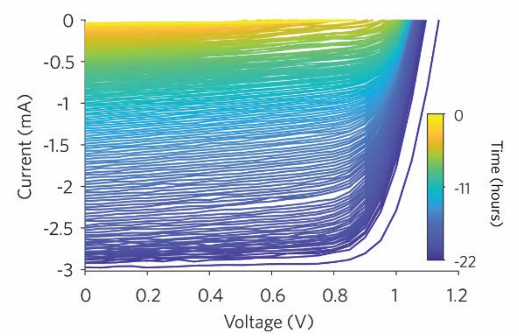

Figure S2 | Current-voltage reverse sweeps of inverted $CH_3NH_3PbI_3$ (MAPI) solar cell under continuous illumination and dry N_2 [Time (hours) < 0].

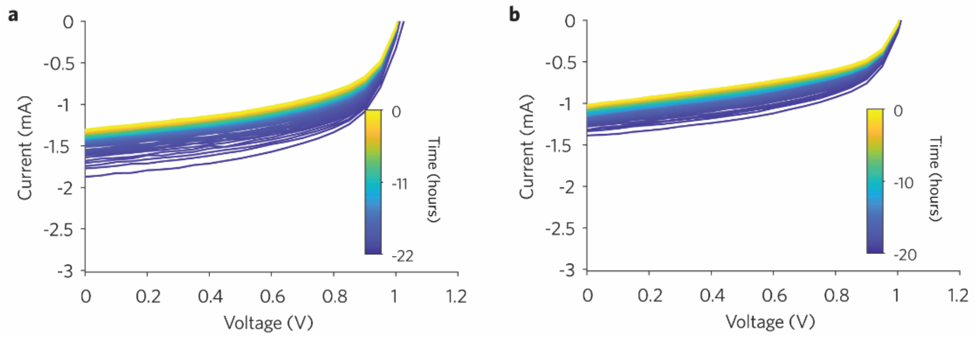

Figure S3 | Current-voltage reverse sweeps of inverted mixed-cation $FA_{0.83}Cs_{0.17}Pb(I_{0.8}Br_{0.2})_3$ solar cell under continuous illumination and dry N_2 [Time (hours) < 0] (a) and dry N_2 (99%) with O_2 (1%) [Time (hours) > 0] (b).

Forward and Reverse Figures-of-Merit

In this section the figures-of-merit are normalized in different ways. In Figure S4 we show FOM extracted from both reverse (see Figure 1 of the manuscript) and forward scans. Here data is normalized twice to the reverse metrics at the beginning of each phase. Reverse (solid lines) and forward (pale lines) are undistinguishable and overlapped when hysteresis is negligible. In Figure S5 we show FOM extracted from the reverse and forward scans, however here data is normalized once to the reverse metrics at the beginning of the stabilization phase. In Figure S6 we show FOM extracted from the forward scans and normalized once to the forward metrics at the beginning of the stabilization phase. In Figure S7 we show FOM extracted from the reverse scans and normalized once to the reverse metrics at the beginning of the stabilization phase.

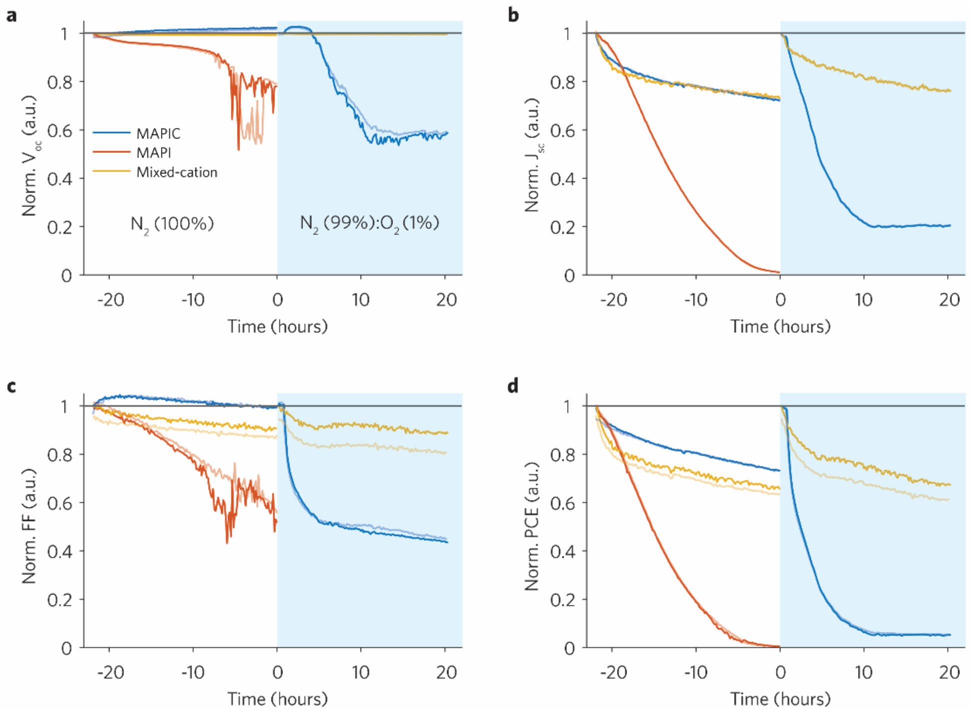

Figure S4 | Evolution of the figures-of-merit (FOM) of inverted MAPIC, MAPI, and mixed-cation PSCs under continuous illumination and dry N_2 (Time < 0) and dry N_2 (99%) with O_2 (1%) (Time > 0). Solid lines correspond to the FOM extracted from the IV reverse sweeps (from 1 V to 0 V). Pale lines represent forward IV sweeps (from 0 V to 1 V). Metrics during stabilization (Time < 0) and stress (Time > 0) phases are normalized to the first recorded value of the reverse metrics during stabilization and stress phases, respectively. Normalized open-circuit voltage V_{oc} (**a**), short-circuit current J_{sc} (**b**), fill factor FF (**c**) and power conversion efficiency PCE (**d**).

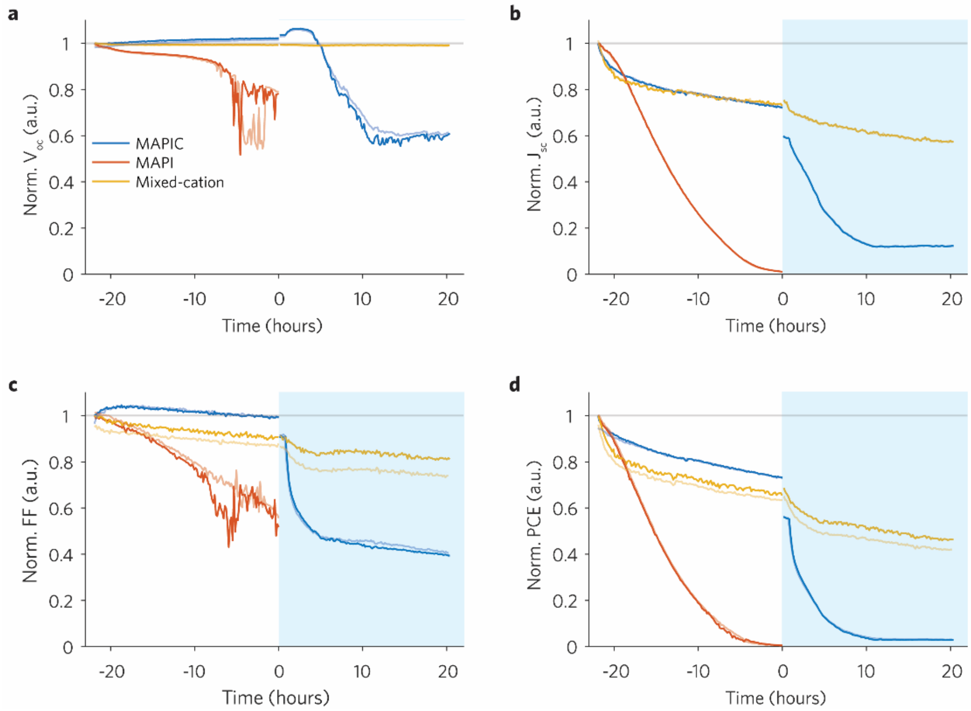

Figure S5 | Evolution of the figures-of-merit (FOM) of inverted MAPIC, MAPI, and mixed-cation PSCs under continuous illumination and dry N_2 (Time < 0) and dry N_2 (99%) with O_2 (1%) (Time > 0). Solid lines correspond to the FOM extracted from the IV reverse sweeps (from 1 V to 0 V). Pale lines represent forward IV sweeps (from 0 V to 1 V). Metrics during stabilization (Time < 0) and stress (Time > 0) phases are normalized once to the first recorded value of the reverse metrics during stabilization. Normalized open-circuit voltage V_{oc} (**a**), short-circuit current J_{sc} (**b**), fill factor FF (**c**) and power conversion efficiency PCE (**d**).

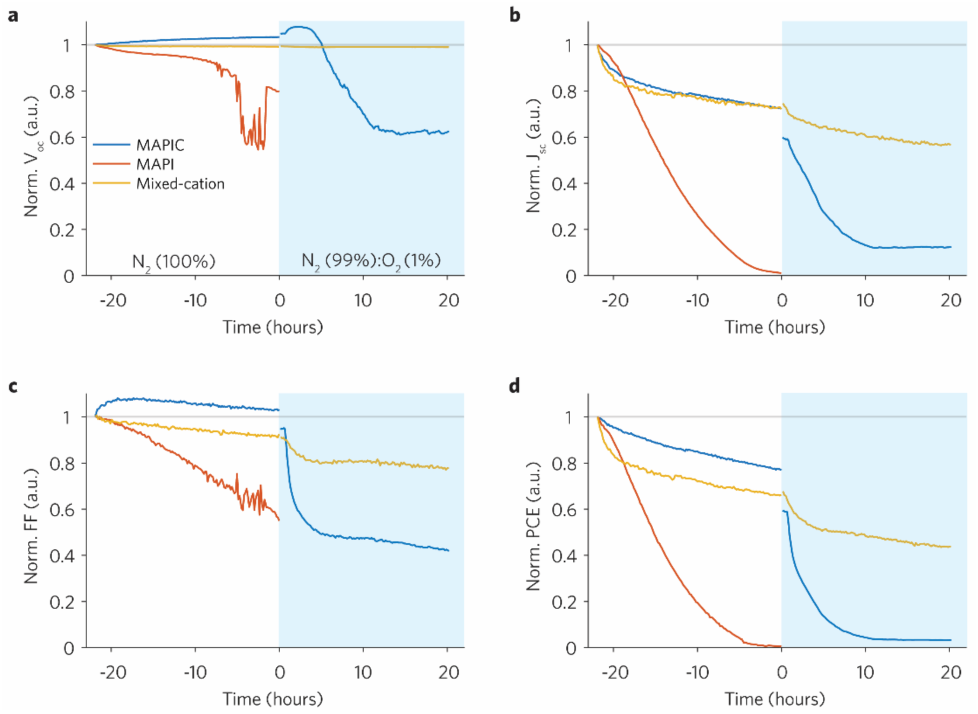

Figure S6 | Evolution of the figures-of-merit (FOM) of inverted MAPIC, MAPI, and mixed-cation PSCs under continuous illumination and dry N_2 (Time < 0) and dry N_2 (99%) with O_2 (1%) (Time > 0). The FOM are extracted from the IV forward sweeps (from 0 V to 1 V). Metrics during stabilization (Time < 0) and stress (Time > 0) phases are normalized once to the first recorded value of the forward metrics during stabilization. Normalized open-circuit voltage V_{oc} (**a**), short-circuit current J_{sc} (**b**), fill factor FF (**c**) and power conversion efficiency PCE (**d**).

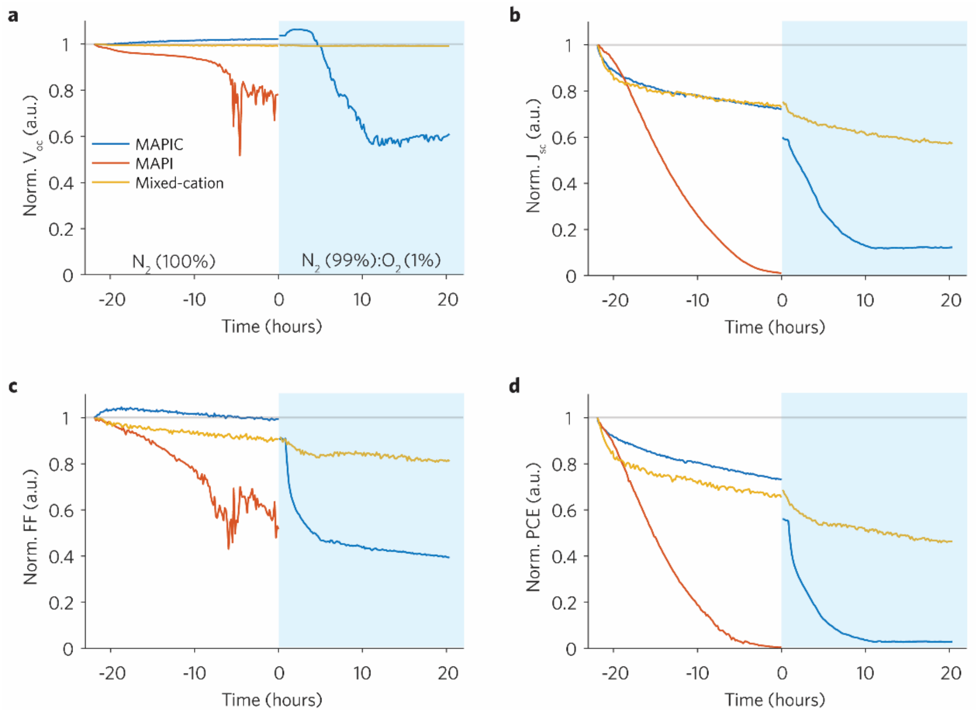

Figure S7 | Evolution of the figures-of-merit (FOM) of inverted MAPIC, MAPI, and mixed-cation PSCs under continuous illumination and dry N_2 (Time < 0) and dry N_2 (99%) with O_2 (1%) (Time > 0). The FOM are extracted from the IV reverse sweeps (from 1 V to 0 V). Metrics during stabilization (Time < 0) and stress (Time > 0) phases are normalized once to the first recorded value of the reverse metrics during stabilization. Normalized open-circuit voltage V_{oc} (**a**), short-circuit current J_{sc} (**b**), fill factor FF (**c**) and power conversion efficiency PCE (**d**).

Hysteresis

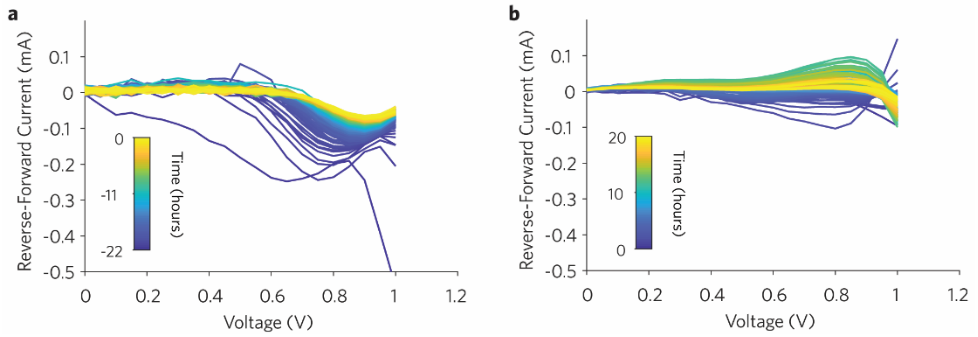

Figure S8 | Reverse minus forward current-voltage sweeps of inverted $CH_3NH_3PbI_{3-x}Cl_x$ (MAPIC) solar cell under continuous illumination and dry N_2 [Time (hours) < 0] (a) and dry N_2 (99%) with O_2 (1%) [Time (hours) > 0] (b).

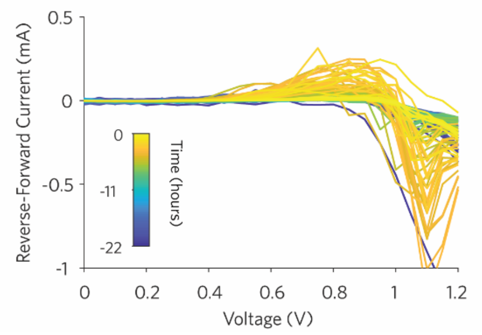

Figure S9 | Reverse minus forward current-voltage sweeps of inverted $CH_3NH_3PbI_3$ (MAPI) solar cell under continuous illumination and dry N_2 [Time (hours) < 0].

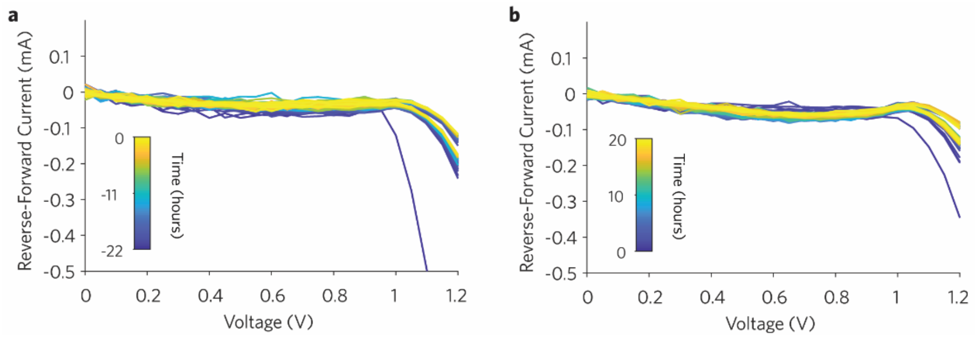

Figure S10 | Reverse minus forward current-voltage sweeps of inverted mixed-cation $FA_{0.83}Cs_{0.17}Pb(I_{0.8}Br_{0.2})_3$ solar cell under continuous illumination and dry N_2 [Time (hours) < 0] (a) and dry N_2 (99%) with O_2 (1%) [Time (hours) > 0] (b).

Transient Photocurrent (TPC)

TPC Data

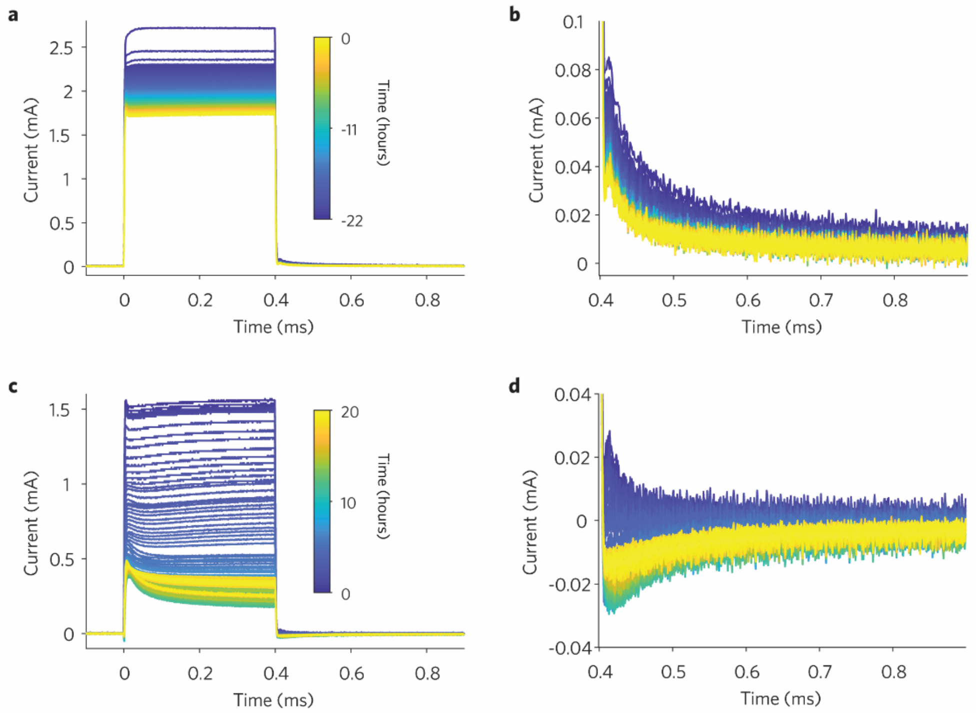

Figure S11 | Transient photocurrent (TPC) measurements of inverted $CH_3NH_3PbI_{3-x}Cl_x$ (MAPIC) solar cell under continuous illumination and dry N_2 [Time (hours) < 0] and dry N_2 (99%) with O_2 (1%) [Time (hours) > 0]. A blue LED light is switched on at Time = 0 ms and off at Time = 0.4 ms, while the photocurrent is continuously monitored. **a**, TPC curves during the stabilization phase under dry nitrogen. **b**, as **a**, zoom in the TPC region after the LED has been switched off. **b**, TPC curves during the stress phase under dry nitrogen (99%) and oxygen (1%). **d**, as **c**, zoom in the TPC region after the LED has been switched off.

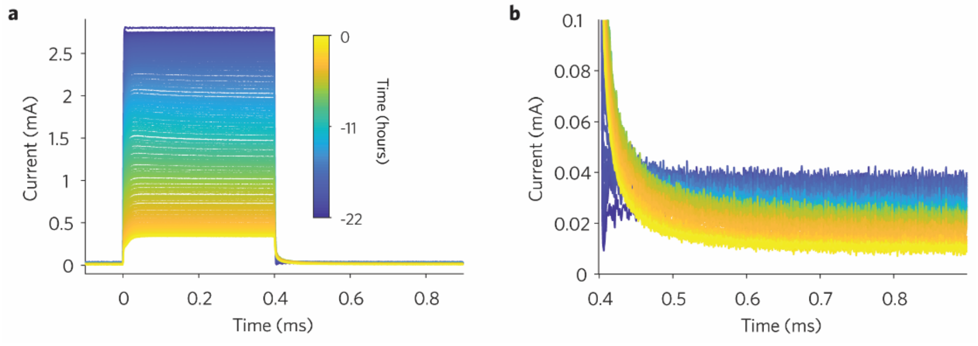

Figure S12 | Transient photocurrent (TPC) measurements of inverted $CH_3NH_3PbI_3$ (MAPI) solar cell under continuous illumination and dry N_2 [Time (hours) < 0]. See caption in Figure S11.

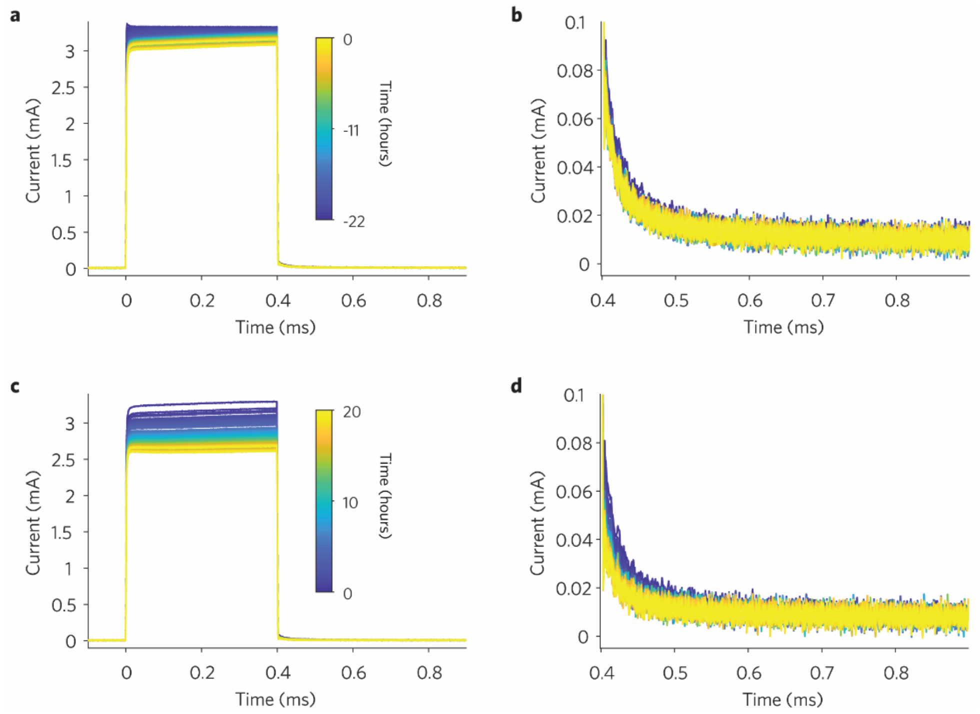

Figure S13 | Transient photocurrent (TPC) measurements of inverted mixed-cation $FA_{0.83}Cs_{0.17}Pb(I_{0.8}Br_{0.2})_3$ solar cell under continuous illumination and dry N_2 [Time (hours) < 0] and dry N_2 (99%) with O_2 (1%) [Time (hours) > 0]. See caption in Figure S11.

Parameters Extracted from the TPC

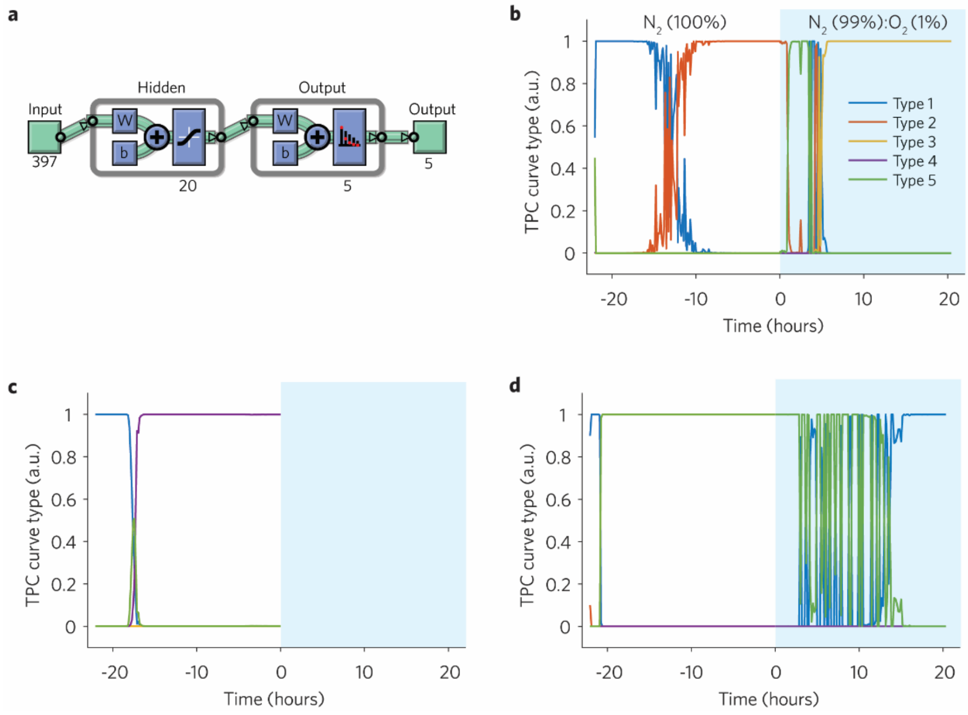

Figure S14 | Classification of TPC behaviour. We first identify five types of TPC behaviour during the LED on interval (see Figure 2a): smooth rise followed by constant current (type 1), fast overshoot followed by linear current increase (type 2), overshoot followed by smooth current decrease (type 3), broad overshoot followed by moderate current decrease (type 4), smooth rise followed by linear current increase (type 5). **a,** Pattern Recognition Neural Network (PRNN) trained with the dataset shown in Figure 2a, with 20 hidden layers (see `patternnet` in MATLAB). Note that in Figure 2a not all type 3 curves used for the training are shown. TPC curve type as clustered by the neural network for MAPIC (**b**), MAPI (**c**), and mixed-cation (**d**). Values equal to 1 indicate that the current TPC curve entirely falls into the indicated cluster. Combinations of curves (at the transition periods) with values between 0 and 1 indicate that the current TPC curve is partially identified as belonging to different clusters.

Table S4 | List of observed TPC curve types.

Solar Cell Type	Observed Curve Type
**MAPIC**	1, 2, 3, 5
**MAPI**	1, 4
**Mixed-cation**	1, 5

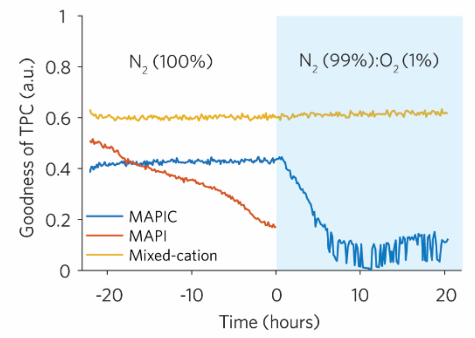

Figure S15 | Goodness of the transient photocurrent (TPC) curves of MAPIC (blue line), MAPI (red line), and mixed-cation [$FA_{0.83}Cs_{0.17}Pb(I_{0.8}Br_{0.2})_3$] (yellow line) solar cells under continuous illumination and dry N_2 [Time (hours) < 0] and dry N_2 (99%) with O_2 (1%) [Time (hours) > 0]. The goodness of TPC estimates how good is the solar cell TPC response to the rectangular LED light pulse discussed in Figure S11-Figure S13, and is calculated as the cross-correlation between the LED light pulse (a rectangular pulse lasting 0.4 ms with 1 as the amplitude) and a normalized TPC curve extracted during the stabilization and stress phases. Note that the time range used for the calculation of the cross-correlation ranges from the moment in which the LED is turned on until 0.401 ms.

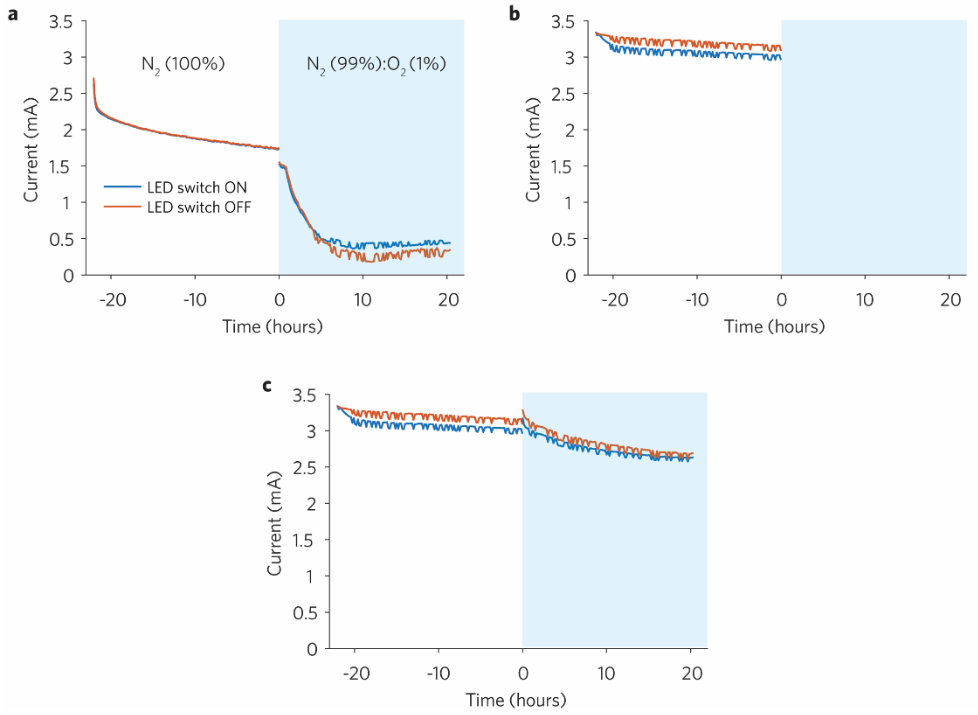

Figure S16 | Evolution of the transient photocurrent (TPC) curves at two points (ON and oFF) for MAPIC (a), MAPI (b), and mixed-cation [$FA_{0.83}Cs_{0.17}Pb(I_{0.8}Br_{0.2})_3$] (c) solar cells under continuous illumination and dry N_2 [Time (hours) < 0] and dry N_2 (99%) with O_2 (1%) [Time (hours) > 0]. LED switch on (blue lines) is the current averaged between 0.004 and 0.014ms. LED switch off (red lines) is the current measured immediately before the LED is turned off at 0.4ms.[2]

Transient Photovoltage (TPV)

Original TPV data

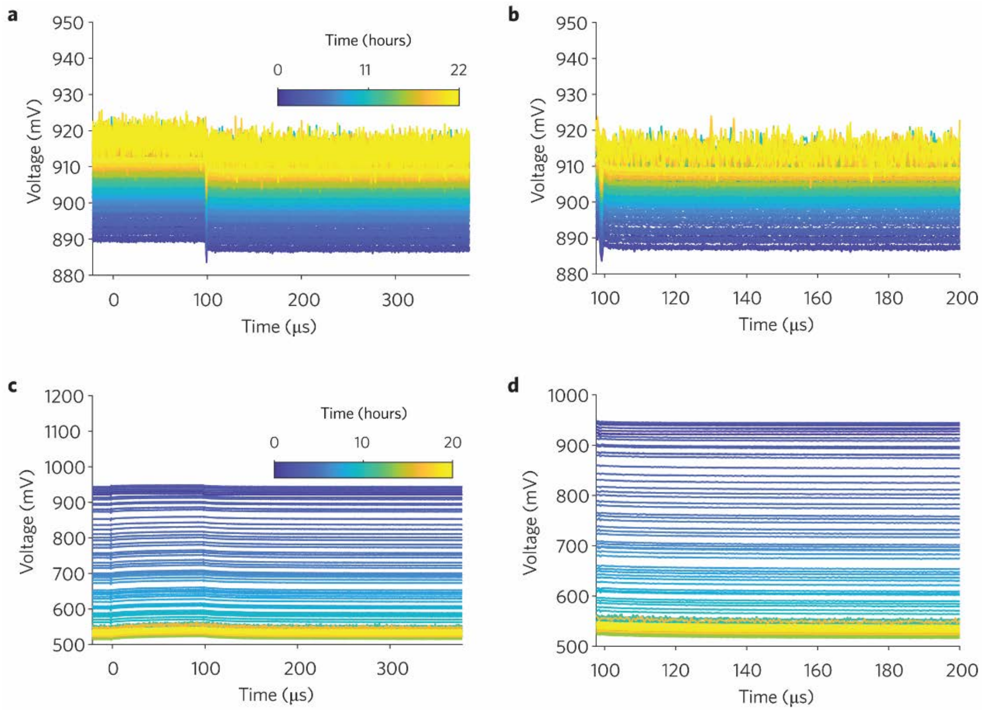

Figure S17 | Transient photovoltage (TPV) measurements of inverted $CH_3NH_3PbI_{3-x}Cl_x$ (MAPIC) solar cell under continuous illumination and dry N_2 [Time (hours) < 0] and dry N_2 (99%) with O_2 (1%) [Time (hours) > 0]. A blue LED light is switched on at Time = -2 ms and off at Time = 98 ms, while the photocurrent is continuously monitored. **a**, TPV curves during the stabilization phase under dry nitrogen. **b**, as **a**, zoom in the TPV region after the LED has been switched off. **b**, TPV curves during the stress phase under dry nitrogen (99%) and oxygen (1%). **d**, as **c**, zoom in the TPV region after the LED has been switched off.

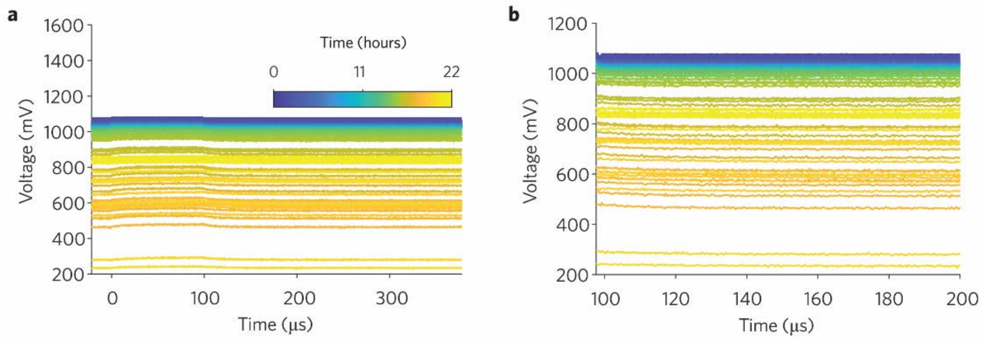

Figure S18 | Transient photocurrent (TPV) measurements of inverted $CH_3NH_3PbI_3$ (MAPI) solar cell under continuous illumination and dry N_2 [Time (hours) < 0]. See caption in Figure S20.

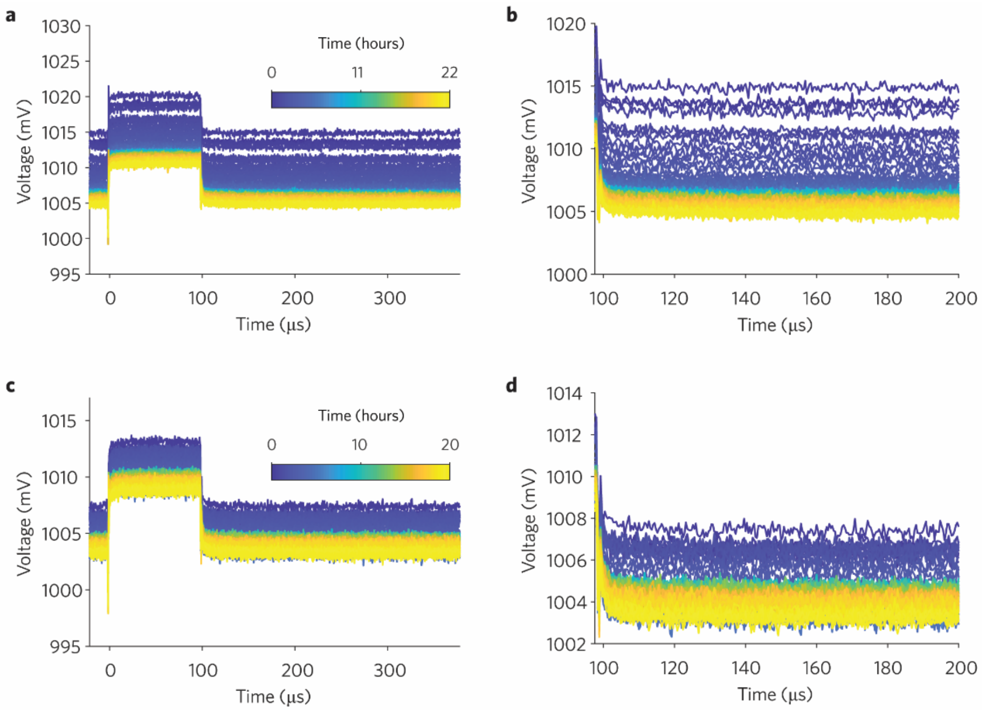

Figure S19 | Transient photocurrent (TPV) measurements of inverted $FA_{0.83}Cs_{0.17}Pb(I_{0.8}Br_{0.2})_3$ solar cell under continuous illumination and dry N_2 [Time (hours) < 0] and dry N_2 (99%) with O_2 (1%) [Time (hours) > 0]. See caption in Figure S20.

'Aligned' TPV data

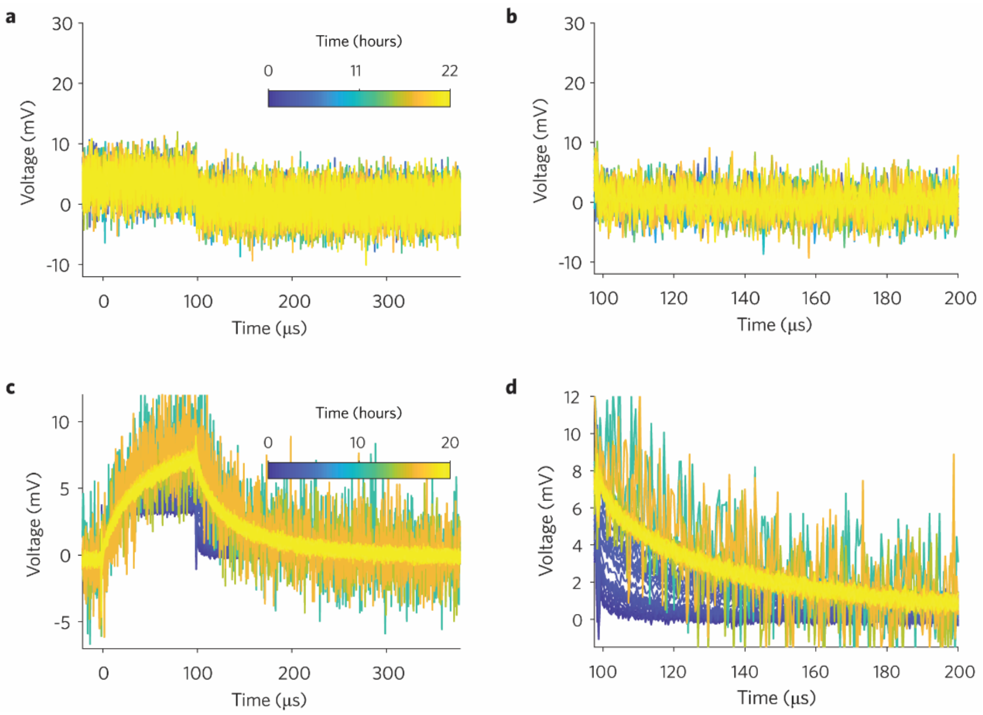

Figure S20 | Aligned transient photovoltage (TPV) measurements of inverted $CH_3NH_3PbI_{3-x}Cl_x$ (MAPIC) solar cell under continuous illumination and dry N_2 [Time (hours) < 0] and dry N_2 (99%) with O_2 (1%) [Time (hours) > 0]. A blue LED light is switched on at Time = -2 ms and off at Time = 98 ms, while the photocurrent is continuously monitored. Each TPV curves is 'aligned' to zero voltage by subtracting from it the average voltage calculated from the last 200 points of the scan (V_{oc}). **a**, TPV curves during the stabilization phase under dry nitrogen. **b**, as **a**, zoom in the TPV region after the LED has been switched off. **b**, TPV curves during the stress phase under dry nitrogen (99%) and oxygen (1%). **d**, as **c**, zoom in the TPV region after the LED has been switched off.

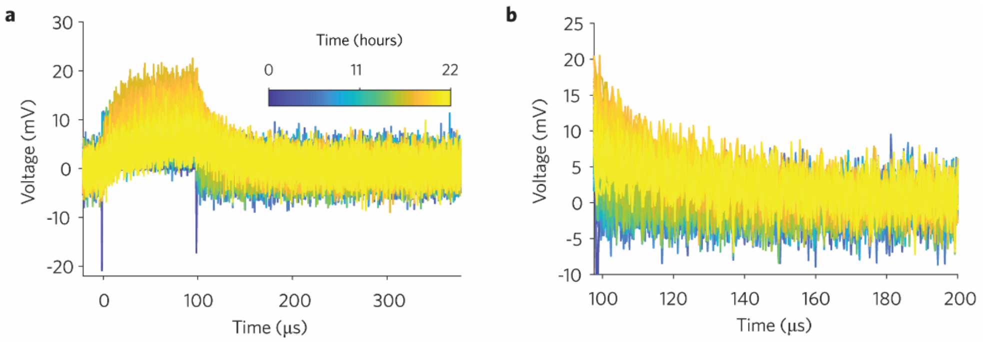

Figure S21 | Aligned transient photocurrent (TPV) measurements of inverted $CH_3NH_3PbI_3$ (MAPI) solar cell under continuous illumination and dry N_2 [Time (hours) < 0]. See caption in Figure S20.

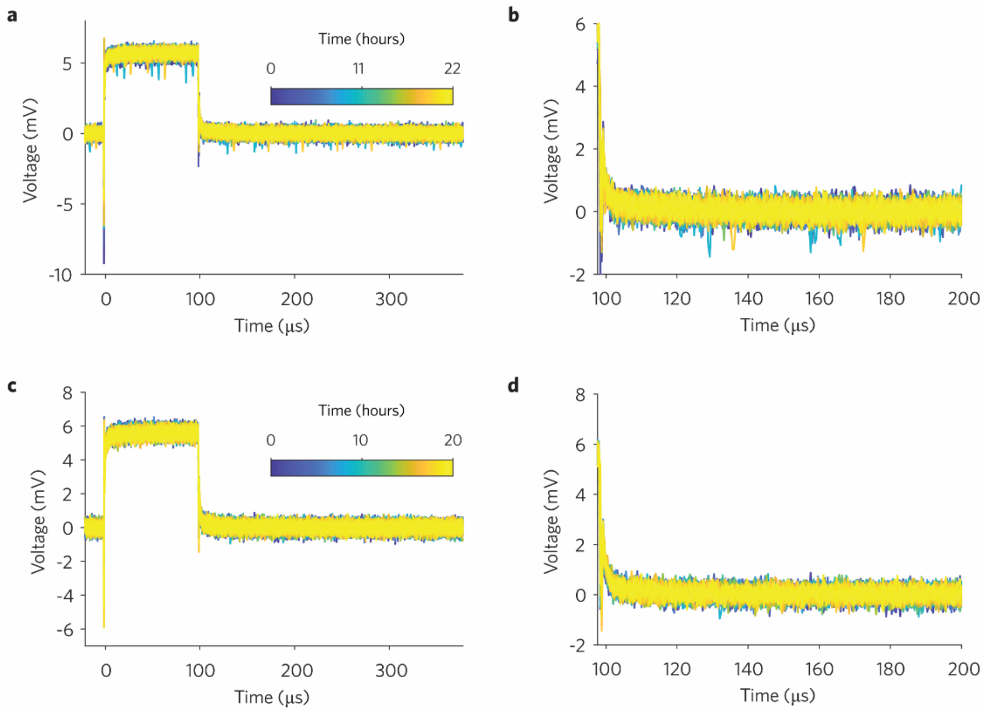

Figure S22 | Aligned transient photocurrent (TPV) measurements of inverted $FA_{0.83}Cs_{0.17}Pb(I_{0.8}Br_{0.2})_3$ solar cell under continuous illumination and dry N_2 [Time (hours) < 0] and dry N_2 (99%) with O_2 (1%) [Time (hours) > 0]. See caption in Figure S20.

Fitting the Exponential Decay

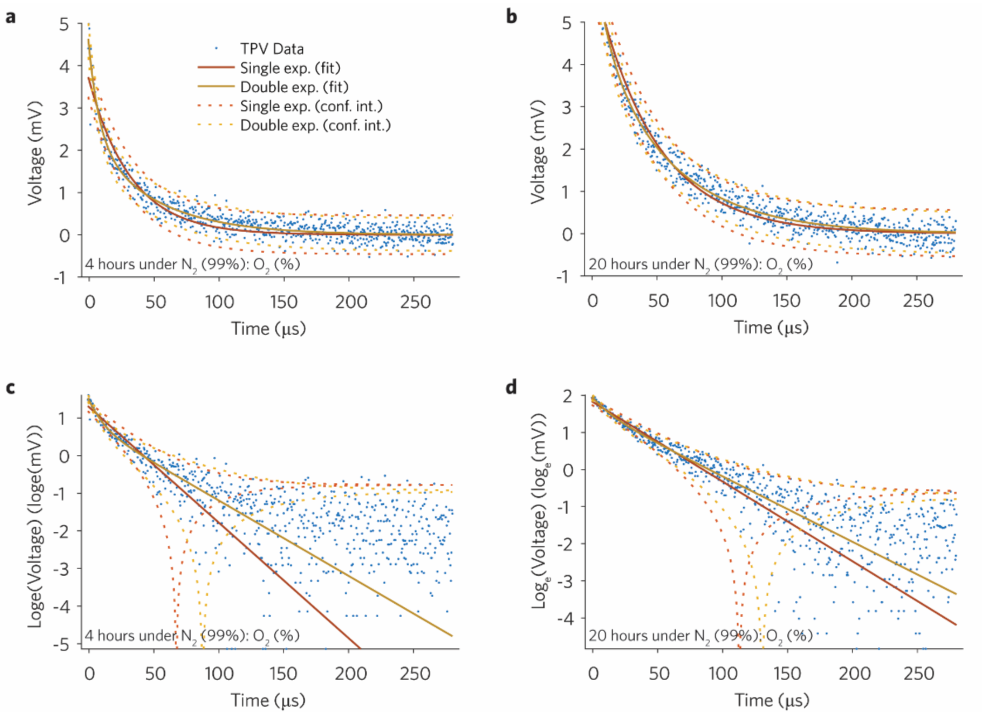

Figure S23 | Examples of single and double exponential fits of the decay period from the (aligned) transient photovoltage (TPV) measurements of inverted $CH_3NH_3PbI_{3-x}Cl_x$ (MAPIC) solar cell under continuous illumination and dry N_2 (99%) with O_2 (1%) [Time (hours) > 0]. Prior to fitting data was 'aligned' (see Figure S20) to zero voltage by subtracting from it the average voltage calculated from the last 200 points of the scan (V_{oc}). The single exponential fitting function is $y = a \times \exp(-x/T)$, where a is a generic constant, x is the Time (μs), and T is a time constant (μs). The double exponential fitting function is $y = a_1 \times \exp(-x/T_1) + a_2 \times \exp(-x/T_2)$, where a_1 and a_2 are a generic constant, x is the Time (μs), and T_1 and T_2 are time constants (μs). Dotted lines indicate the 95% confidence intervals calculated with the fit procedure. **a**, TPV decay and single/double exponential fits (after 4 hours exposure to N_2 (99%): O_2 (1%)). **b**, as in **a** after 20 hours exposure. **c**, As in **a** with the plots of $\log_e$ TPV and $\log_e$ of the fits (after 4 hours). **d**, As in **b** with the plots of $\log_e$ TPV and $\log_e$ of the fits (after 4 hours). Note that in the $\log_e$ plots negative data cannot be shown.

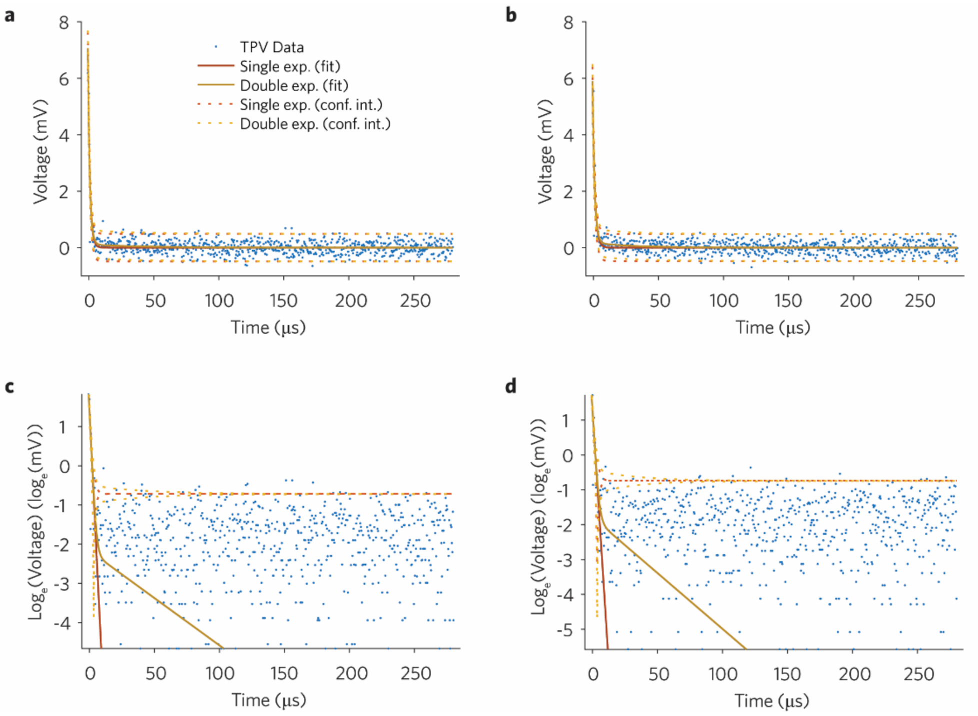

Figure S24 | Examples of single and double exponential fits of the decay period from the transient photovoltage (TPV) measurements of inverted $FA_{0.83}Cs_{0.17}Pb(I_{0.8}Br_{0.2})_3$solar cells under continuous illumination and dry N_2 (99%) with O_2 (1%) [Time (hours) > 0]. See caption in Figure S22.

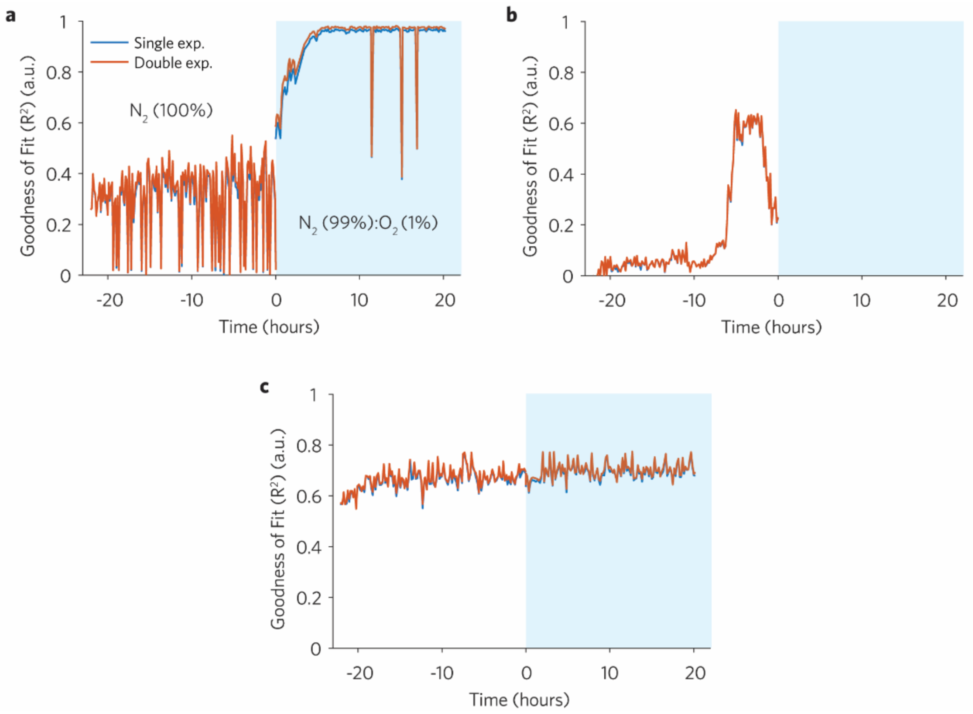

Figure S25 | Goodness of fit (R^2) calculated from the single and double exponential fits of the decay period from the transient photovoltage (TPV) measurements of MAPIC (a), MAPI (b), and mixed-cation [$FA_{0.83}Cs_{0.17}Pb(I_{0.8}Br_{0.2})_3$] (c) solar cells under continuous illumination and dry N_2 (Time < 0) and dry N_2 (99%) with O_2 (1%) (Time > 0). A value of the goodness of fit equal to 1 indicates a perfect fit. The noisy portions in **a** are due to noisy TPV curves.

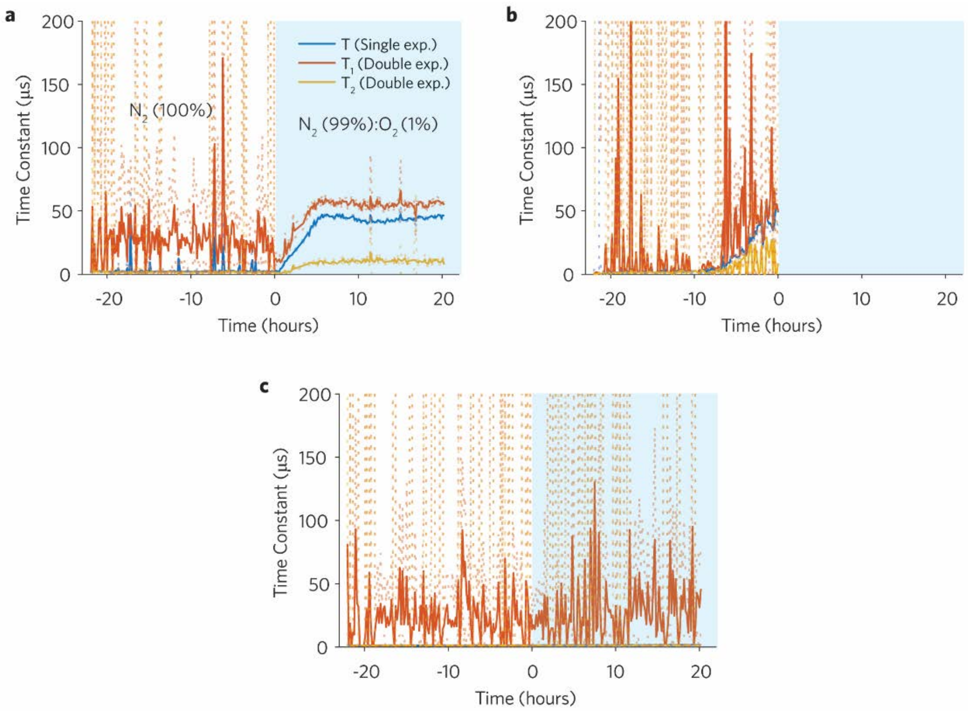

Figure S26 | Fitted time constants from the single and double exponential fits of the decay period from the transient photovoltage (TPV) measurements of MAPIC (a), MAPI (b), and mixed-cation [$FA_{0.83}Cs_{0.17}Pb(I_{0.8}Br_{0.2})_3$] (c) solar cells under continuous illumination and dry N_2 (Time < 0) and dry N_2 (99%) with O_2 (1%) (Time > 0). The dotted lines represent the 95% confidence intervals of the fitted line. The single exponential fitting function is $y = a \times \exp(-x/T)$, where a is a generic constant, x is the Time (μs), and T is a time constant (μs). The double exponential fitting function is $y = a_1 \times \exp(-x/T_1) + a_2 \times \exp(-x/T_2)$, where a_1 and a_2 are a generic constant, x is the Time (μs), and T_1 and T_2 are time constants (μs). Note that the time constants are meaningful only when considered together with the fitted amplitude parameters.

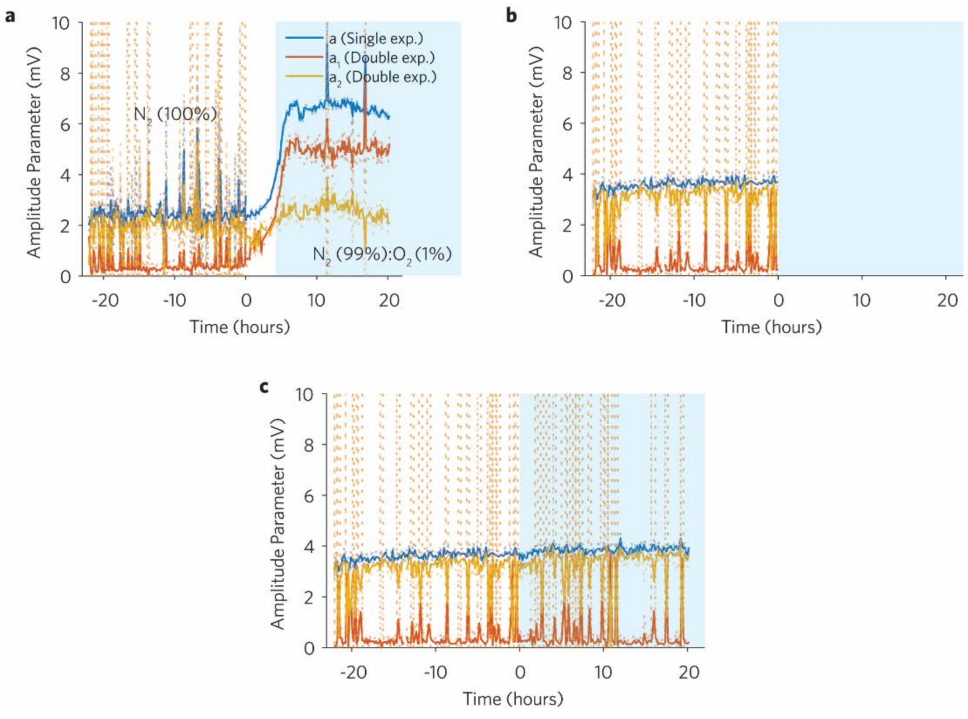

Figure S27 | Fitted amplitude parameter from the single and double exponential fits of the decay period from the transient photovoltage (TPV) measurements of MAPIC (a), MAPI (b), and mixed-cation [$FA_{0.83}Cs_{0.17}Pb(I_{0.8}Br_{0.2})_3$] (c) solar cells under continuous illumination and dry N_2 (Time < 0) and dry N_2 (99%) with O_2 (1%) (Time > 0). The fitted amplitudes are a, a1, a2 (see Figure S26).

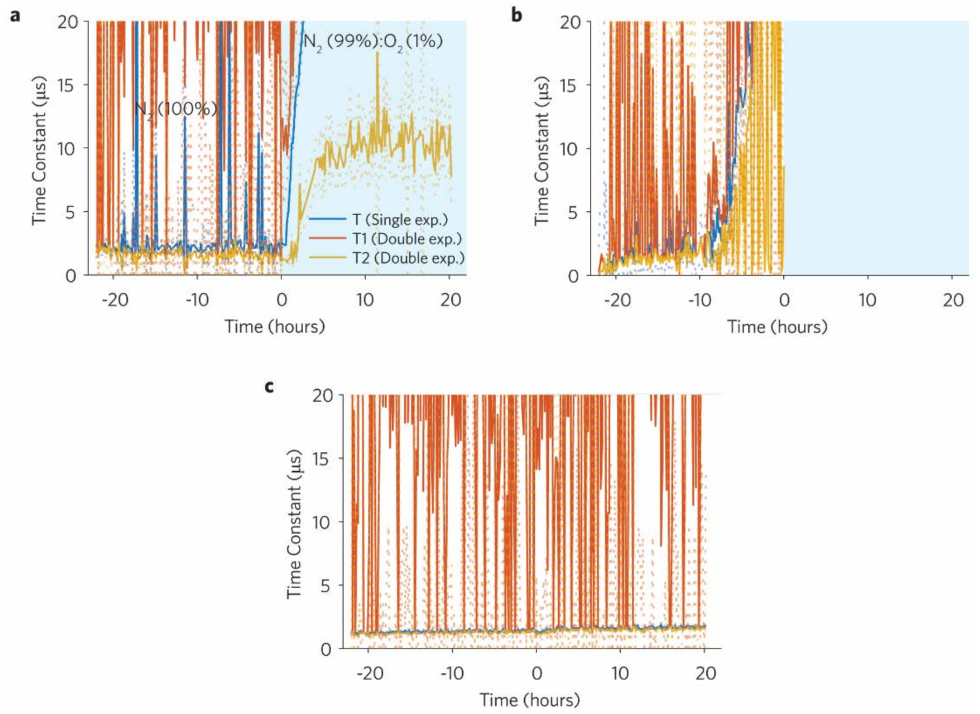

Figure S28 | Fitted time constants, zoom of Figure S26.

Series and Parallel Resistance Extracted from IV Sweeps

We estimate parallel (R_p) and series (R_s) resistances from the IV curves under illumination from the inverse of the slopes of tangent lines at the short circuit and open circuit points, respectively (Figure S29)

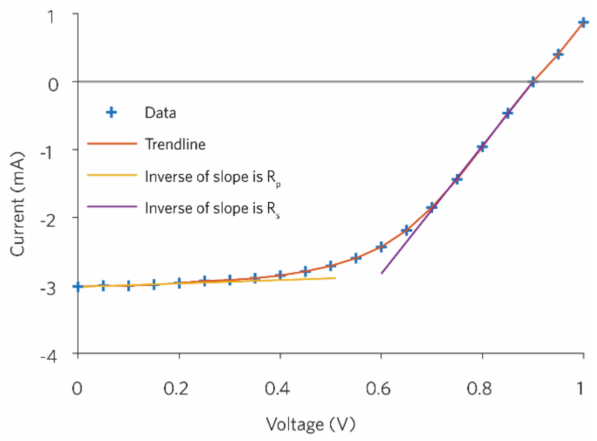

Figure S29 | Illustration of how parallel and series resistance are extracted.

Figure S30 show plots of R_p and R_s (from reverse and forward sweeps) for the MAPIC, MAPI, and mixed-cation solar cells during ageing. We note that a more accurate estimate of series and parallel resistances should be performed on IV curves under dark conditions, which were not measured throughout the ageing.

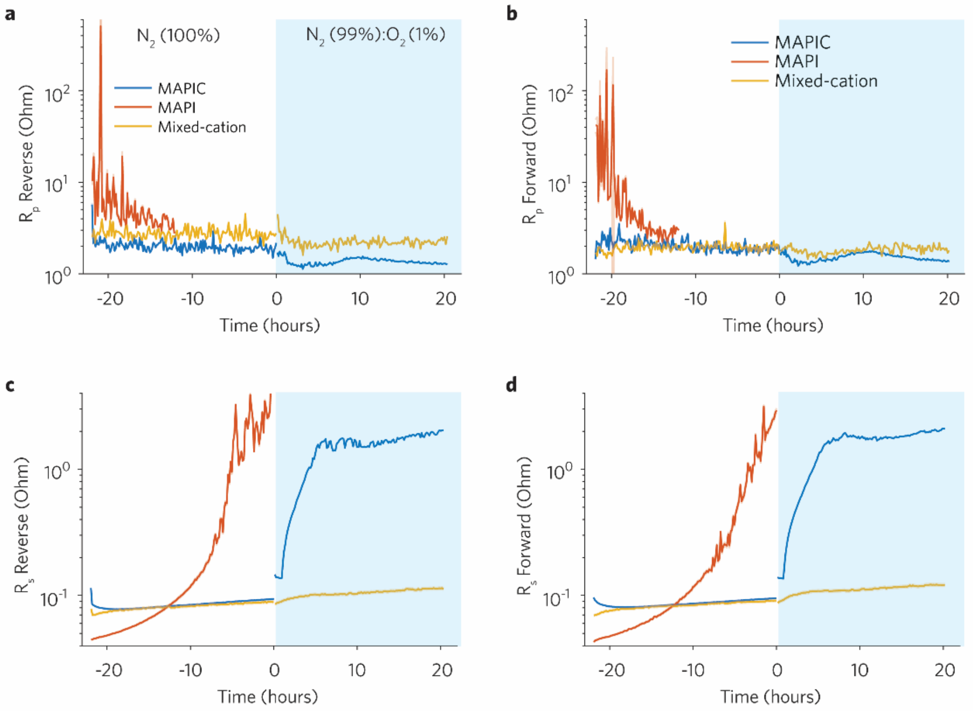

Figure S30 | Evolution of the parallel (R_p) and series (R_s) resistance of inverted MAPIC, MAPI, and mixed-cation PSCs under continuous illumination and dry N_2 (Time < 0) and dry N_2 (99%) with O_2 (1%) (Time > 0). (**a**) R_p extracted from reverse sweeps. (**b**) R_p extracted from forward sweeps. (**c**) R_s extracted from reverse sweeps. (**d**) R_s extracted from forward sweeps. Note that in (a) and (b) R_s values for the MAPI solar cell for Time > -13 hours have been removed as results were unreliable due to the IV curve shape (see Figure S2). Semi-transparent lines, when visible, indicate the relative error on R_s or R_p (calculated from the error on the fitted slope parameter).

Light Dependant IV Sweeps Metrics

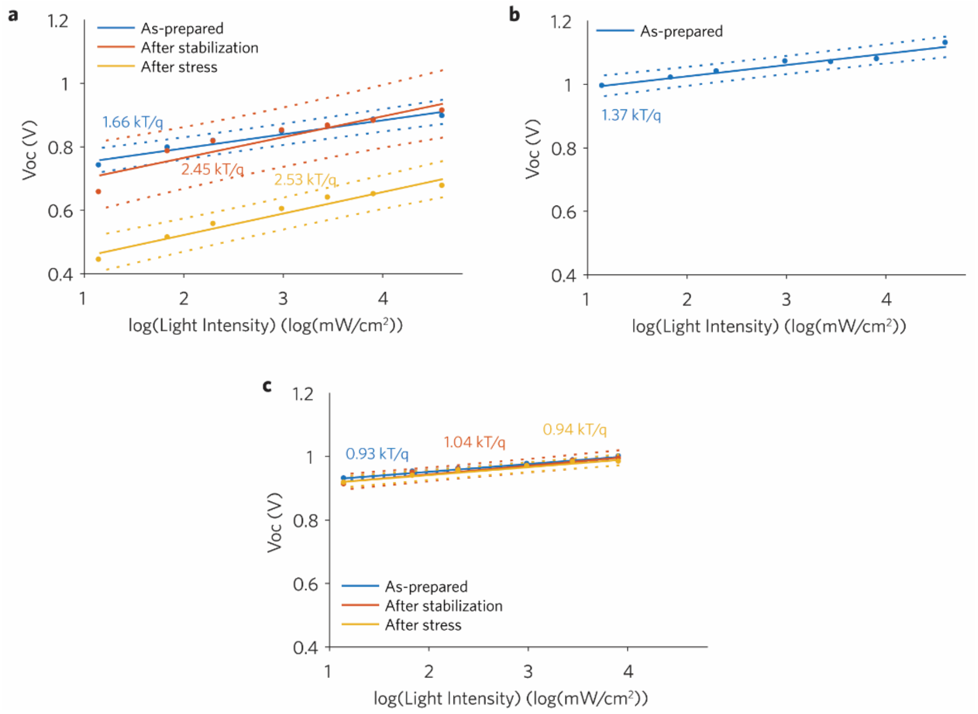

Figure S31 | Ideality factor *n*: plots and fits of Voc vs log(Light Intensity). 'As-prepared' indicates that the measurements were performed before the stabilization phase (N_2 (100%) with continuous light). 'After stabilization' indicates that the measurements were performed after the stabilization phase. 'After stress' indicates that the measurements were performed after the stress phase (N_2 (99%): O_2(1%) with continuous light). Dots indicate data points. Straight lines indicate the fitting line n × kT/q × log(Light Intensity) + *b*, where *b* is a generic constant, *k* is the Boltzman constant, *T* is the temperature during the measurement in Kelvin, *q* is the elementary charge in Coulombs, and *n* is the ideality factor. Dotted lines represent the 95% confidence intervals calculated with the fit. The numbers in the graph represent *n* as n × kT/q. **a**, MAPIC. **b**, MAPI. **c**, $FA_{0.83}Cs_{0.17}Pb(I_{0.8}Br_{0.2})_3$.

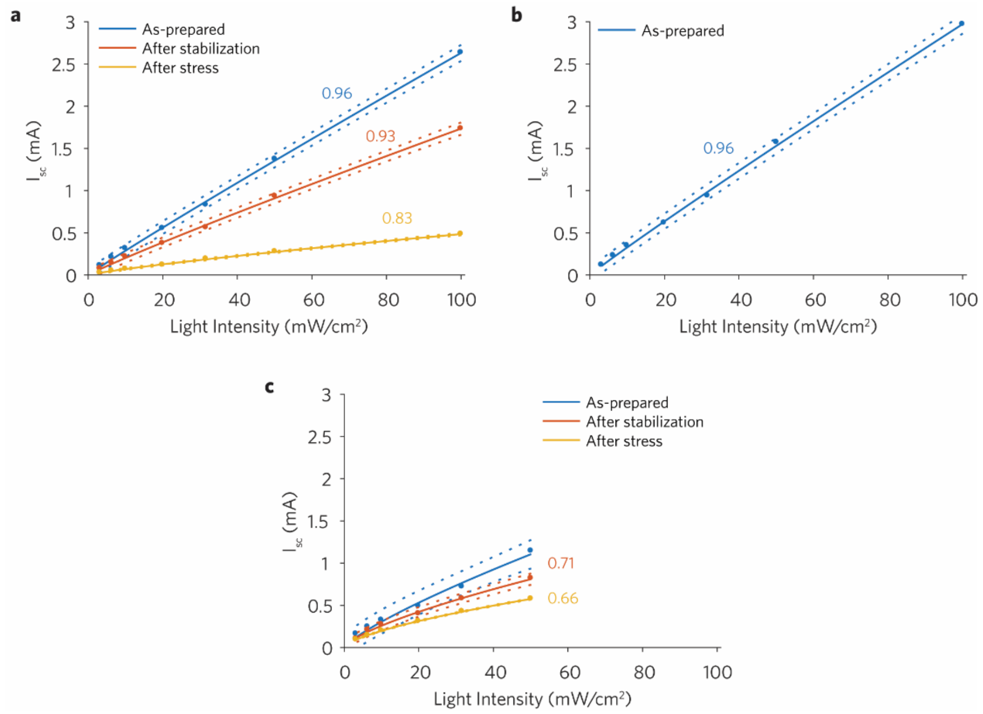

Figure S32 | Alpha factor: plots and fits of I_{sc} vs Light Intensity. 'As-prepared' indicates that the measurements were performed before the stabilization phase (N_2 (100%) with continuous light). 'After stabilization' indicates that the measurements were performed after the stabilization phase. 'After stress' indicates that the measurements were performed after the stress phase (N_2 (99%): O_2(1%) with continuous light). Dots indicate data points. Straight lines indicate the fitting power law b × (Light Intensity)^{alpha}, where *b* is a generic constant. Dotted lines represent the 95% confidence intervals calculated with the fit. The numbers in the graph represent alpha. **a**, MAPIC. **b**, MAPI. **c**, $FA_{0.83}Cs_{0.17}Pb(I_{0.8}Br_{0.2})_3$.

Light Dependant TPC

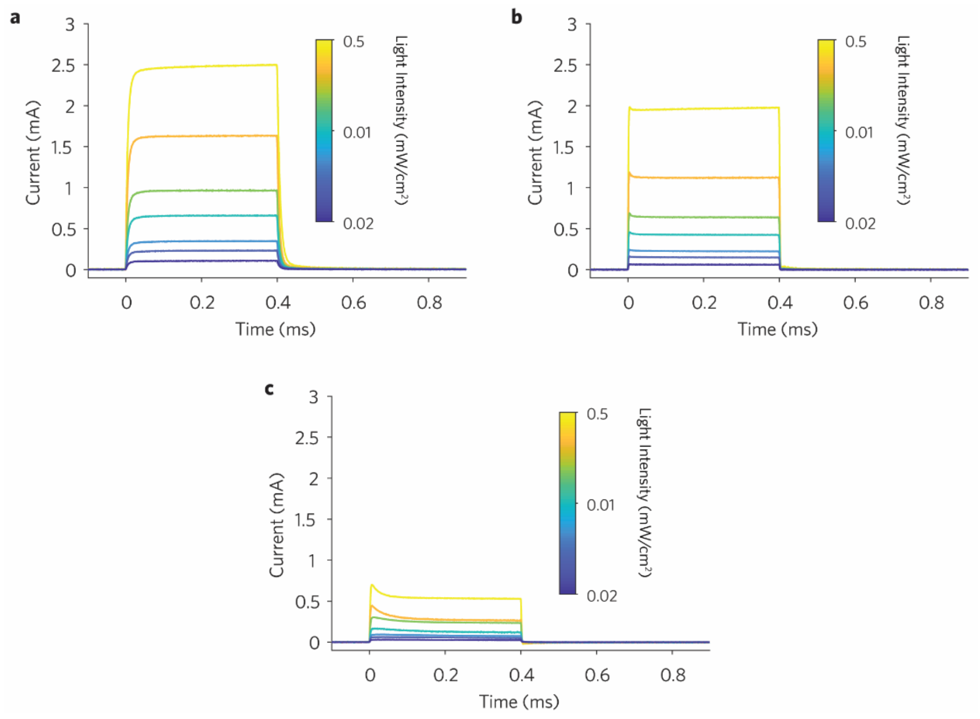

Figure S33 | TPC curves vs Light Intensity for as-prepared (a), after stabilization (b), after stress (c) MAPIC.

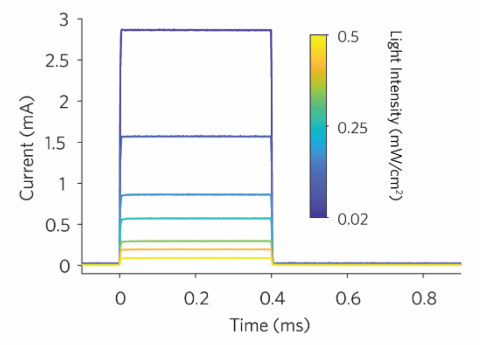

Figure S34 | TPC curves vs Light Intensity for as-prepared (a), after stabilization (b), after stress (c) MAPI.

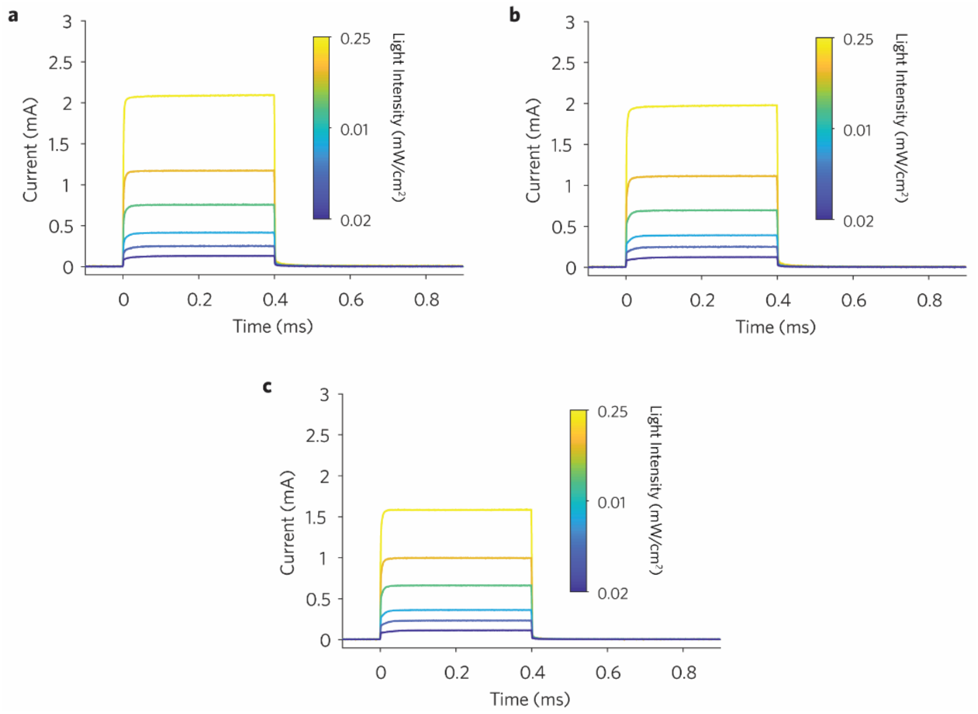

Figure S35 | TPC curves vs Light Intensity for as-prepared (a), after stabilization (b), after stress (c) $FA_{0.83}Cs_{0.17}Pb(I_{0.8}Br_{0.2})_3$.

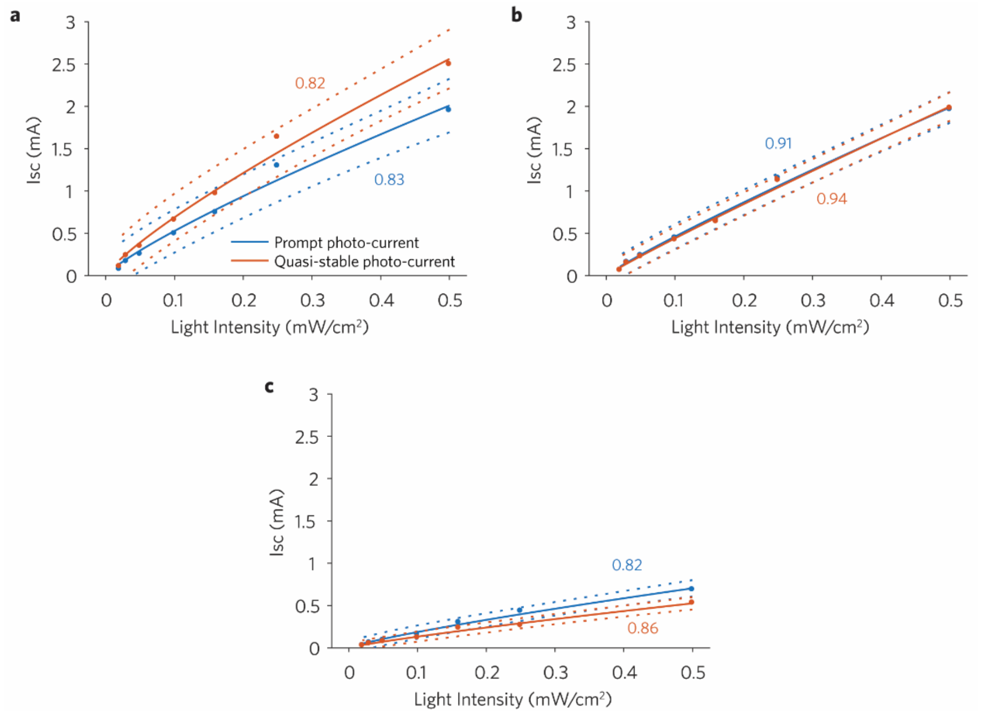

Figure S36 | Alpha factor: plots and fits of prompt photocurrent and quasi-stable photocurrent from TPC curves vs Light Intensity for as-prepared (a), after stabilization (b), after stress (c) MAPIC . Dots indicate data points. Straight lines indicate the fitting power law b × (Light Intensity)^alpha, where *b* is a generic constant. Dotted lines represent the 95% confidence intervals calculated with the fit. The numbers in the graph represent alpha.

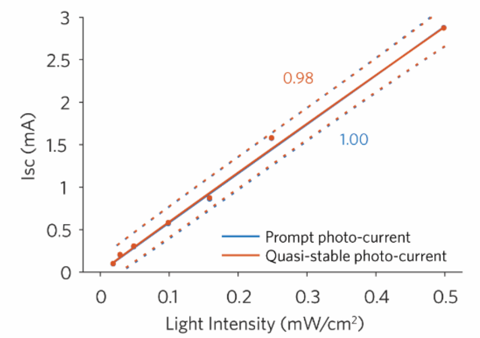

Figure S37 | Alpha factor: plots and fits of prompt photocurrent and quasi-stable photocurrent from TPC curves vs Light Intensity for as-prepared (a), after stabilization (b), after stress (c) MAPI . Dots indicate data points. Straight lines indicate the fitting power law b × (Light Intensity)^alpha, where *b* is a generic constant. Dotted lines represent the 95% confidence intervals calculated with the fit. The numbers in the graph represent alpha.

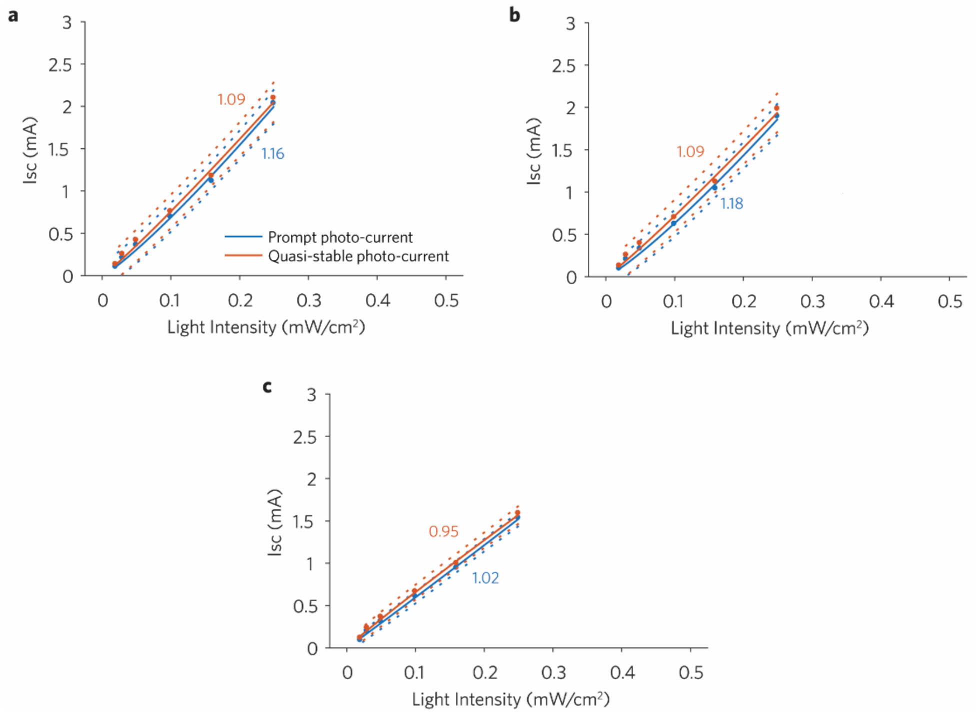

Figure S38 | Alpha factor: plots and fits of prompt photocurrent and quasi-stable photocurrent from TPC curves vs Light Intensity for as-prepared (a), after stabilization (b), after stress (c) $FA_{0.83}Cs_{0.17}Pb(I_{0.8}Br_{0.2})_3$. Dots indicate data points. Straight lines indicate the fitting power law b × (Light Intensity)^{alpha}, where *b* is a generic constant. Dotted lines represent the 95% confidence intervals calculated with the fit. The numbers in the graph represent alpha.

Light Dependant TPV

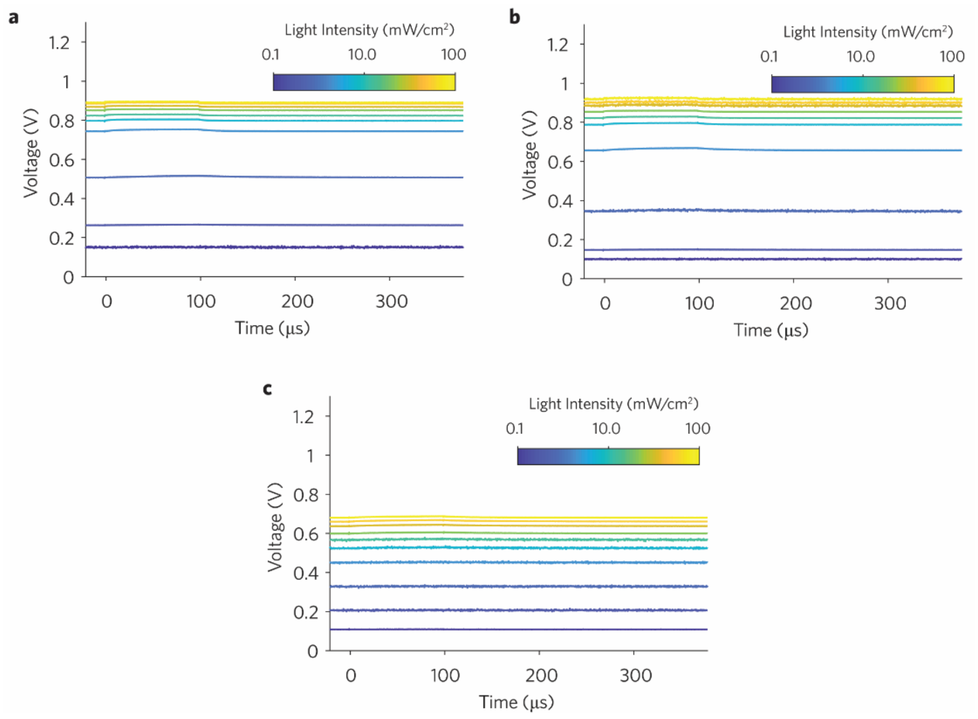

Figure S39 | TPV curves vs Light Intensity for as-prepared (a), after stabilization (b), after stress (c) MAPIC.

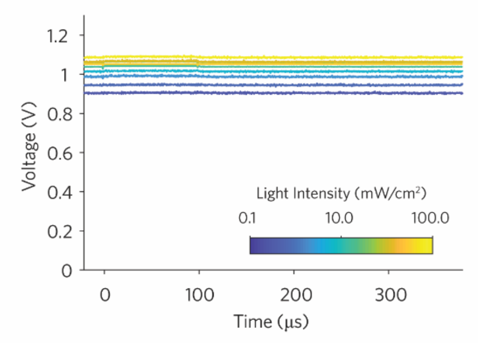

Figure S40 | TPV curves vs Light Intensity for as-prepared (a), after stabilization (b), after stress (c) MAPI .

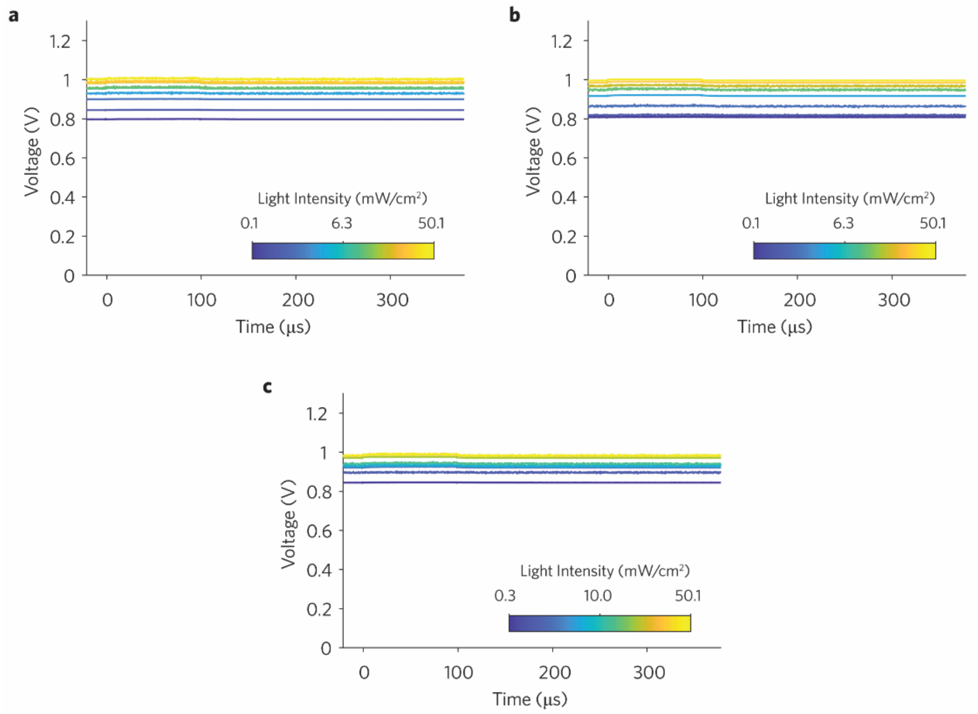

Figure S41 | TPV curves vs Light Intensity for as-prepared (a), after stabilization (b), after stress (c) $FA_{0.83}Cs_{0.17}Pb(I_{0.8}Br_{0.2})_3$.

TPV Decay Time Constant vs V_{oc}

Single Exponential Fit

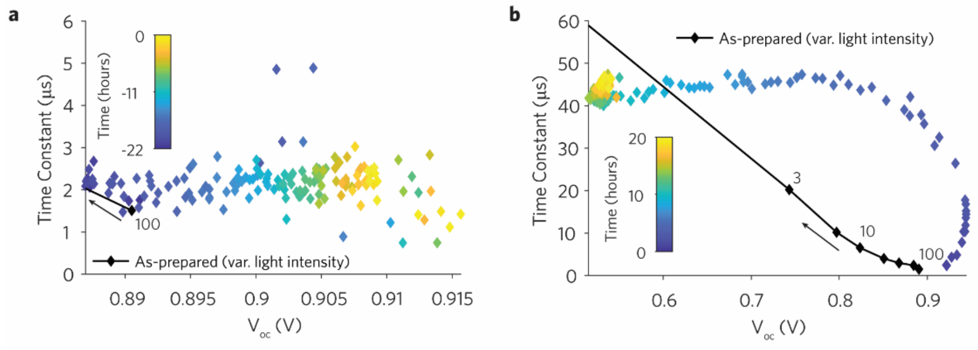

Figure S42 | Fitted time constant (single exponential fit) vs V_{oc} extracted from the transient photovoltage (TPV) measurements of MAPIC. a, Stabilization phase (Time (hours) < 0). **b**, Stress phase (Time (hours) > 0). The ideal behaviour profile behaviour is plotted as a black line and is represented by the time constant vs V_{oc} extracted from TPV traces measured at different light intensities before the stabilization phase (as-prepared devices). Time constant is extracted with the single exponential fit of the TPV decay. Numbers in the figure indicate the light intensity in mW/cm^2.

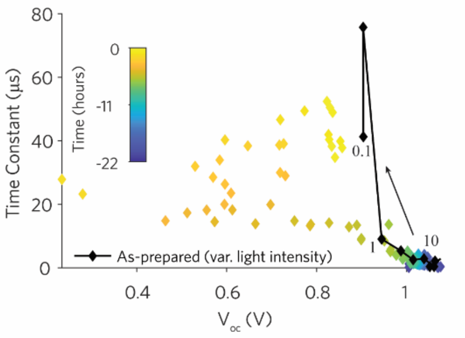

Figure S43 | Fitted time constant (single exponential fit) vs V_{oc} extracted from the transient photovoltage (TPV) measurements of MAPI during the stabilization phase (Time (hours) < 0). See caption in Figure S42.

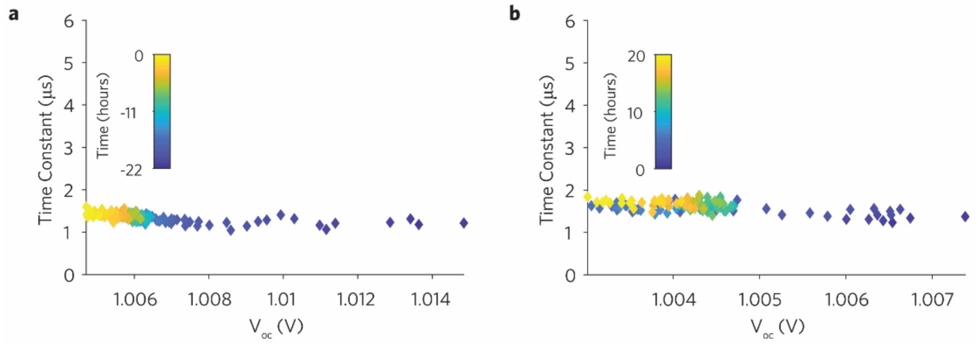

Figure S44 | Fitted time constant (single exponential fit) vs V_{oc} extracted from the transient photovoltage (TPV) measurements of $[FA_{0.83}Cs_{0.17}Pb(I_{0.8}Br_{0.2})_3]$. a, Stabilization phase (Time (hours) < 0). **b**, Stress phase (Time (hours) > 0). See caption in Figure S42.

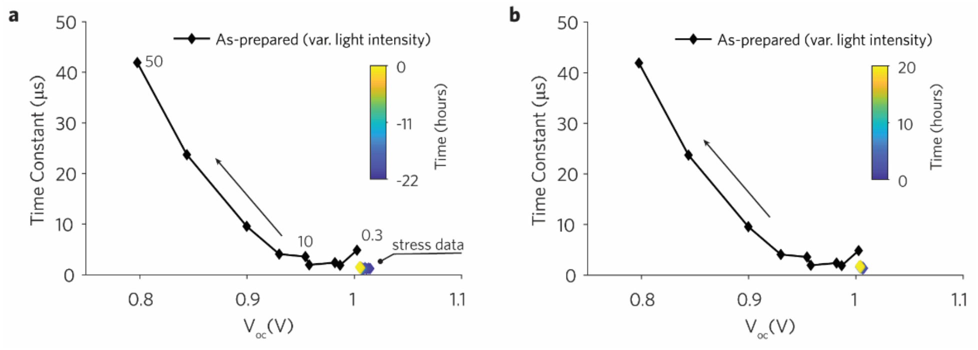

Figure S45 | Fitted time constant (single exponential fit) vs V_{oc} extracted from the transient photovoltage (TPV) measurements of $[FA_{0.83}Cs_{0.17}Pb(I_{0.8}Br_{0.2})_3]$. a, Stabilization phase (Time (hours) < 0). **b**, Stress phase (Time (hours) > 0). The V_{oc} range has been extended to show that the ideal behaviour (black line) is outside the range of the TPV measurements during the stabilization and stress phases. See caption in Figure S42.

Double Exponential Fit

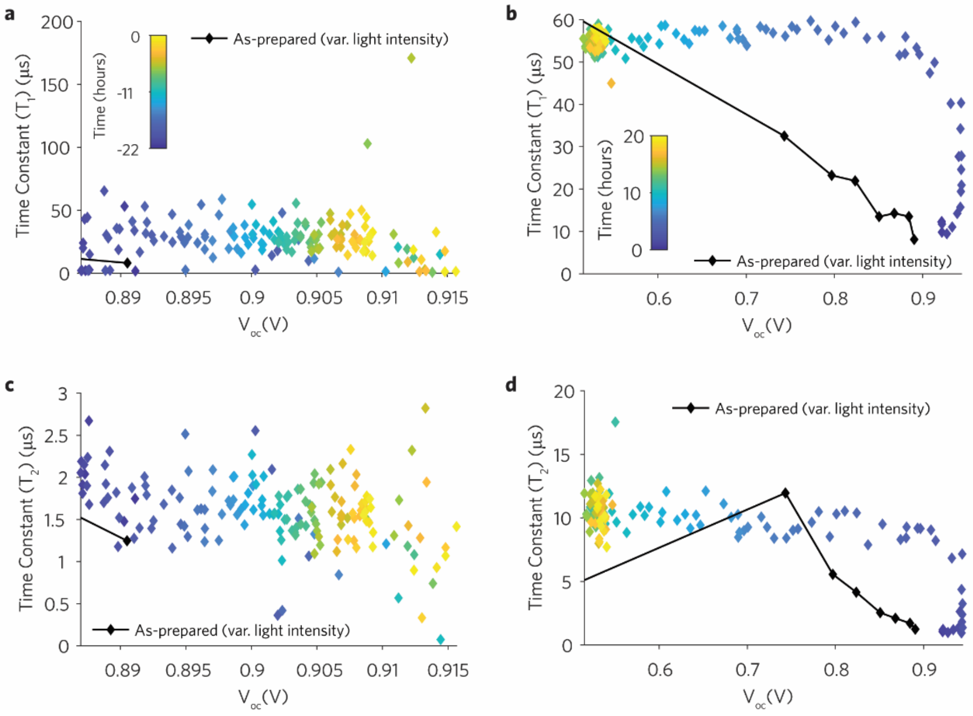

Figure S46 | Fitted time constants (double exponential fit) vs V_{oc} extracted from the transient photovoltage (TPV) measurements of MAPIC. a, Stabilization phase (Time (hours) < 0). **b**, Stress phase (Time (hours) > 0). The ideal behaviour profile behaviour is plotted as a black line and is represented by the time constant (T_1 or T_2) vs the V_{oc} extracted from TPV traces measured at different light intensities before the stabilization phase (as-prepared devices). Time constants are extracted from the double exponential fit of the TPV decay. Numbers in the figure indicate the light intensity in mW/cm^2.

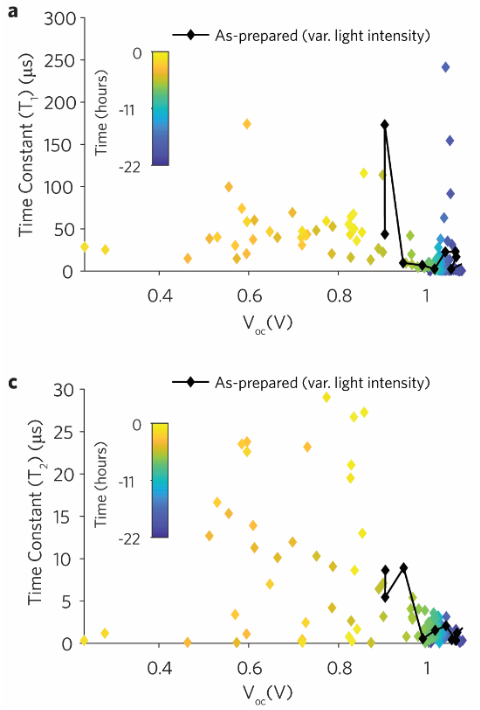

Figure S47 | Fitted time constants (double exponential fit) vs V_{oc} extracted from the transient photovoltage (TPV) measurements of MAPbI. a, Time constant T_1 during the stabilization phase (Time (hours) < 0). **b,** Time constant T_2 during the stabilization phase (Time (hours) > 0). See caption in Figure S46.

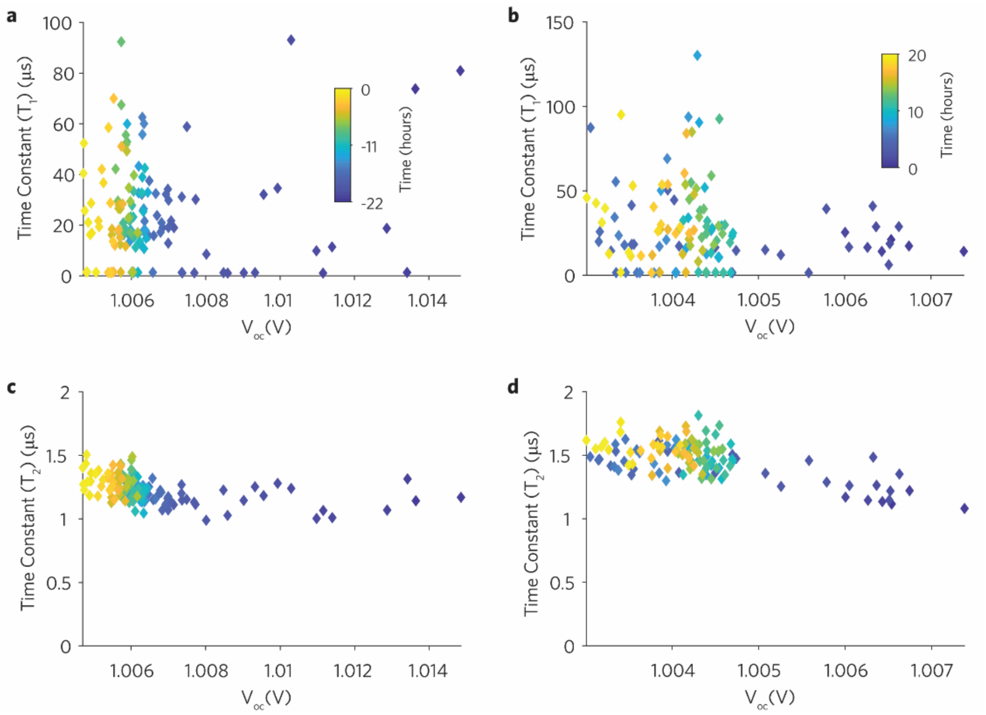

Figure S48 | Fitted time constants (double exponential fit) vs V_{oc} extracted from the transient photovoltage (TPV) measurements of [$FA_{0.83}Cs_{0.17}Pb(I_{0.8}Br_{0.2})_3$]. a, Time constant T_1 during the stabilization phase (Time (hours) < 0). **b,** Time constant T_1 during the stress phase (Time (hours) > 0). **c,** Time constant T_2 during the stabilization phase. **d,** Time constant T_2 during the stress phase. See caption in Figure S46. As shown in Figure S44-Figure S45.

References

1 Wang, J. T.-W., Wang, Z., Pathak, S., Zhang, W., deQuilettes, D. W., Wisnivesky-Rocca-Rivarola, F., Huang, J., Nayak, P. K., Patel, J. B., Mohd Yusof, H. A., Vaynzof, Y., Zhu, R., Ramirez, I., Zhang, J., Ducati, C., Grovenor, C., Johnston, M. B., Ginger, D. S., Nicholas, R. J. & Snaith, H. J. Efficient perovskite solar cells by metal ion doping. *Energy Environ. Sci.* **9**, 2892-2901, (2016).

2 Pearson, A. J., Eperon, G. E., Hopkinson, P. E., Habisreutinger, S. N., Wang, J. T.-W., Snaith, H. J. & Greenham, N. C. Oxygen Degradation in Mesoporous Al2O3/CH3NH3PbI3-xClx Perovskite Solar Cells: Kinetics and Mechanisms. *Adv. Energy Mater.* **6**, 1600014-n/a, (2016).
